# Oral SMEDDS promotes lymphatic transport and mesenteric lymph nodes target of chlorogenic acid for effective T-cell antitumor immunity

**DOI:** 10.1136/jitc-2021-002753

**Published:** 2021-07-16

**Authors:** Jun Ye, Yue Gao, Ming Ji, Yanfang Yang, Zhaohui Wang, Baolian Wang, Jing Jin, Ling Li, Hongliang Wang, Xiaoyan Xu, Hengfeng Liao, Chunfang Lian, Yaqi Xu, Renjie Li, Tong Sun, Lili Gao, Yan Li, Xiaoguang Chen, Yuling Liu

**Affiliations:** 1 State Key Laboratory of Bioactive Substance and Function of Natural Medicines, Institute of Materia Medica, Chinese Academy of Medical Sciences & Peking Union Medical College, Beijing, People's Republic of China; 2 Beijing Key Laboratory of Drug Delivery Technology and Novel Formulation, Institute of Materia Medica, Chinese Academy of Medical Sciences & Peking Union Medical College, Beijing, People's Republic of China

**Keywords:** brain neoplasms, drug evaluation, preclinical, immunity, immunotherapy, immunomodulation

## Abstract

**Background:**

Mesenteric lymph nodes (MLNs) are critical draining lymph nodes of the immune system that accommodate more than half of the body’s lymphocytes, suggesting their potential value as a cancer immunotherapy target. Therefore, efficient delivery of immunomodulators to the MLNs holds great potential for activating immune responses and enhancing the efficacy of antitumor immunotherapy. Self-microemulsifying drug delivery systems (SMEDDS) have attracted increasing attention to improving oral bioavailability by taking advantage of the intestinal lymphatic transport pathway. Relatively little focus has been given to the lymphatic transport advantage of SMEDDS for efficient immunomodulators delivery to the MLNs. In the present study, we aimed to change the intestinal lymphatic transport paradigm from increasing bioavailability to delivering high concentrations of immunomodulators to the MLNs.

**Methods:**

Chlorogenic acid (CHA)-encapsulated SMEDDS (CHA-SME) were developed for targeted delivery of CHA to the MLNs. The intestinal lymphatic transport, immunoregulatory effects on immune cells, and overall antitumor immune efficacy of CHA-SME were investigated through in vitro and in vivo experiments.

**Results:**

CHA-SME enhanced drug permeation through intestinal epithelial cells and promoted drug accumulation within the MLNs via the lymphatic transport pathway. Furthermore, CHA-SME inhibited tumor growth in subcutaneous and orthotopic glioma models by promoting dendritic cell maturation, priming the naive T cells into effector T cells, and inhibiting the immunosuppressive component. Notably, CHA-SME induced a long-term immune memory effect for immunotherapy.

**Conclusions:**

These findings indicate that CHA-SME have great potential to enhance the immunotherapeutic efficacy of CHA by activating antitumor immune responses.

## Background

An ever-growing understanding of immune system regulation and activation in tumors has driven the exploration of targeted and immune-based cancer therapies. Cancer immunotherapeutic strategies require engagement with immune cells, which are unevenly distributed in the body and concentrated in the lymph nodes and lymphoid organs.^1^ Lymph nodes play a critical role in the adaptive immune system, which organizes lymphocyte (LYM) accumulation, activation, and proliferation.^2^ Therefore, strategies that deliver immunomodulators directly to lymph nodes provide an opportunity to modify the adaptive immune response, thereby improving cancer immunotherapeutic outcomes.^3^ However, because of the structure and anatomy of lymph nodes as well as the distinct localization and migration of the different cell types within them, it is challenging to access the lymphatics via traditional drug delivery routes via the blood circulation.^2 4^

To overcome this challenge, various nanocarriers, including nanoparticles, micelles, liposomes, and nanoemulsions, have been designed for targeted delivery of immunomodulators to the lymph nodes.^4–7^ Self-microemulsifying drug delivery systems (SMEDDS), which are considered ideal alternative oral drug delivery carriers, have attracted increasing attention owing to their potential to promote drug transport into the intestinal lymphatics.^8–11^ The effectiveness of SMEDDS is highlighted by several successful, marketed products, such as Sandimmune Neoral.^12 13^ Lymphatic transport allows extraordinary gains in bioavailability and efficacy through avoidance of first-pass hepatic metabolism and drug preservation in lymphatic tissues, mainly mesenteric lymph nodes (MLNs), which have proven critical in the improvement of oral bioavailability by SMEDDS.^9 10 14^ As up to 50% of LYM are concentrated within the intestinal lymphatic system, promoting drug delivery to the intestinal lymphatics via SMEDDS is expected to result in the co-localization of high concentrations of drugs and LYM in a single compartment, leading to enhanced drug access to and activity on LYM.^15^ Substantial in vivo studies have shown that SMEDDS improve oral bioavailability, which has been attributed to its lymphatic transport-promoting feature.^9–11 16 17^ Another interesting potential advantage of SMEDDS in lymphatic transport, which, to the best of our knowledge, has not been attracted sufficient attention in cancer immunotherapy, is their ability to promote the targeting of immunomodulators to LYM and thus facilitate direct action of immunomodulators on immune cells.

Chlorogenic acid (CHA), a phenolic acid found in traditional Chinese medicine herbs, has multiple beneficial properties, especially, antitumor activity mediated by immunomodulatory pathways.^18–22^ We previously found that CHA functions as an antitumor immunomodulator that promotes the polarization of tumor-associated macrophages from the M2 to the M1 phenotype, thereby modulating the tumor microenvironment and inhibiting the growth of glioblastoma.^20 23^ In addition, CHA enhances the transcription of immune factors, such as interleukin 2 receptor (IL-2R) and interferon-γ (IFN-γ), promotes the proliferation and activation of T cells, natural killer cells, and macrophages, and suppresses tumor growth.^19 24^ Currently, CHA is being evaluated in phase II and III clinical trials in advanced glioblastoma patients (NCT03758014) as a class 1 innovative small-molecular natural drug. The beneficial antitumor immunomodulatory effects on tumor-associated macrophages and T cells and the encouraging phase I clinical outcomes make CHA an attractive candidate for cancer immunotherapy. However, an important drawback of the regimen applied in ongoing clinical trials is that CHA has to be administered daily by intramuscular injection for months due to its rapid clearance in vivo, leading to poor patient compliance.^25^ Potential strategies to improve the stability and bioavailability of CHA have been widely explored in the context of drug absorption into the blood, but have been less robustly evaluated in MLNs to date.^26 27^ As a hydrophilic small-molecule compound, CHA is poorly absorbed through the small intestine barrier and is widely metabolized by the gut microflora, resulting in low oral bioavailability and lymphatic accumulation.^28^ The lymphatic transport strategy by encapsulating oral SMEDDS with CHA may improve CHA stability and permeability in the gastrointestinal tract, promote targeted delivery of CHA to the MLNs via lymphatic transport pathway, thus facilitate the immunomodulatory action of CHA on immune cells concentrated within MLNs, especially, T cells.

**Figure 1:**
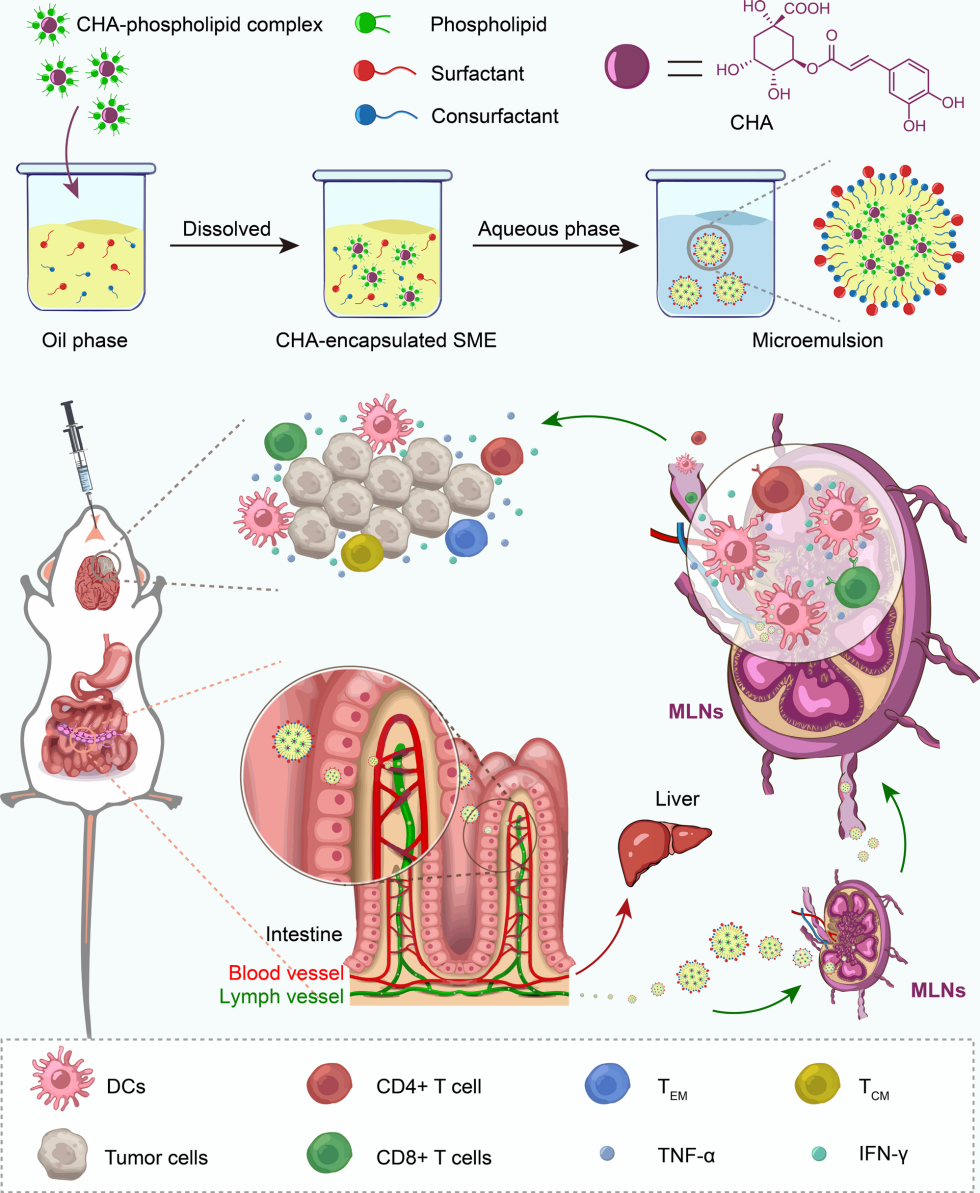
Schematic illustration of the preparation, the intestinal lymphatic transport, and immunomodulatory action of CHA-SME on immune cells.

Glioblastoma is the most common and aggressive type of primary malignant brain tumor, with a median survival of <2 years. The current standard care approach for glioblastoma includes surgery, radiation, and chemotherapy with temozolomide (TMZ). However, the development of resistance and common toxicities of TMZ are major problems that limits the effectiveness of chemotherapy.^29^ Therefore, there remains a significant unmet need for improved therapeutic strategies, and there have been substantial efforts exploring novel approaches, especially cancer immunotherapy. The introduction of immune checkpoint inhibitors has dramatically improved the antitumor outcomes for some advanced tumors, leading to the provision of new research directions for the glioblastoma treatment.^30^ However, the survival and other outcomes have not improved significantly in several recent clinical trials with immune checkpoint inhibitors against glioblastoma.^31 32^ The poor penetration of immune checkpoint inhibitors across blood-brain barrier and drug resistance are supposed to compromise their therapeutic efficiency in combating glioblastoma.^32 33^ In this study, we developed an oral CHA-encapsulated SMEDDS (CHA-SME) for targeted delivery of CHA to the MLNs via lymphatic transport for glioblastoma immunotherapy with circumventing poor penetration and drug resistance in immune checkpoint blockade therapy of glioblastoma. Transepithelial transport of CHA-SME across the intestinal tract, targeted CHA delivery to the MLNs via lymphatic transport, the subsequent immunomodulatory effect on LYM, including dendritic cells (DCs) and T cells, and the potential of this approach to enhance immunotherapeutic efficacy were systematically investigated in vitro and in vivo. This study provided a potential new approach for MLNs-targeted cancer immunotherapy that efficiently delivers CHA to the MLNs via lymphatic transport, facilitates the immunomodulatory action of CHA on immune cells, and enhances the antitumor immune efficacy of CHA by regulating the antitumor immune function of T cells, with negligible systemic toxicity (figure 1).

## Material and methods

### Materials

CHA was kindly provided by Sichuan Jiuzhang Biotech (Chengdu, Sichuan, China). Soybean phospholipid S75 was purchased from Shanghai Tywei Pharmaceutical (Shanghai, China). Labrasol and Transcutol HP were purchased from Gattefossé (Saint-Priest, Lyon, France). TMZ and cycloheximide were obtained from J&K Scientific (Beijing, China). Coumarin-6 (Cou-6), methyl-β-cyclodextrin (M-β-CD), chlorpromazine, and lipopolysaccharide (LPS) were purchased from Sigma-Aldrich (Saint Louis, MO, USA). Genistein and amiloride hydrochloride were supplied by the National Institutes for Food and Drug Control (Beijing, China). Cholesterol was purchased from A.V.T. Pharmaceutical (Shanghai, China). Brefeldin A, monensin, and nocodazole were purchased from Beyotime Biotechnology (Shanghai, China). Bafilomycin A1 was purchased from Dalian Meilun Biotechnology (Liaoning, China). 1,1-dioctadecyl-3,3,3,3-tetramethylindotricarbocyanine iodide (DiR) was purchased from AAT Bioquest (Sunnyvale, California, USA). Dulbecco’s modified Eagle’s medium (DMEM), MEM, and fetal bovine serum (FBS) were purchased from Thermo Fisher Scientific (Waltham, MA, USA). Fluorochrome-labeled anti-mouse monoclonal antibodies (CD45, CD3, CD4, CD8, CD44, CD62L, CD11c, class I major histocompatibility complex (MHC I), MHC II, CD40, CD80, and CD86) and bead-based LEGENDplex kits were purchased from BioLegend (San Diego, California, USA). All other organic reagents were of analytical grade and were purchased from Sinopharm Chemical Reagent (Shanghai, China).

### Animals and cell culture

Female ICR mice (18–20 g) and male Sprague-Dawley (SD) rats (180–220 g) were supplied by Beijing Vital River Laboratory Animal Technology (Beijing, China). Beagle dogs (8–11 kg) were supplied by Beijing Marshall Biotechnology (Beijing, China).

Caco-2 human colon carcinoma cells were obtained from the Cell Resource Center, Peking Union Medical College (Beijing, China). C6 rat glioma cells were a kind gift from Prof. Zhonggao Gao (Institute of Materia Medica, Peking Union Medical College, Beijing, China). Caco-2 and C6 cells were cultured in MEM or DMEM supplemented with 10% FBS, 100 U/mL penicillin, and 100 µg/mL streptomycin in a humidified atmosphere of 5% CO_2_ at 37°C.

### Preparation and characterization of CHA-SME

The CHA-phospholipid complex was prepared with CHA and soybean phospholipid S75 using a solvent evaporation method. Briefly, CHA and phospholipids at a mass ratio of 1:2.2 were dissolved in absolute ethanol and stirred for 15 min at room temperature. Subsequently, the organic solvent was evaporated under vacuum using a rotary evaporator. The CHA-phospholipid complex was collected, dried under vacuum, weighed (XS105DU; Mettler-Toledo GmbH, Zurich, Switzerland), and stored at –20°C until use.

To prepare SMEDDS loaded with CHA-phospholipid complex (CHA-SME), an accurately weighed quantity of CHA-phospholipid complex was mixed with a homogenous solution of ethyl oleate, Labrasol, and Transcutol HP at a mass ratio of 1:3:2. The mixture was shaken vigorously at room temperature to obtain a transparent solution. CHA-SME loaded with a fluorescent dye, such as Cou-6 or DiR, was prepared using a similar procedure, except that Cou-6 or DiR was dissolved along with the CHA-phospholipid complex.

For in vitro characterization of CHA-SME, 100 µL of CHA-SME was slowly dropped into 10 mL of ultrapure water under magnetic stirring to form a microemulsion. Mean particle size, size distribution, and zeta potential of the microemulsion were measured by dynamic light scattering (DLS) method. The morphology of the microemulsion was observed by transmission electron microscopy (TEM) (H-7650; Hitachi, Tokyo, Japan), atomic force microscopy (AFM) (Dimension Icon; Bruker, Karlsruhe, Baden-Württemberg, Germany), and cryogenic TEM (cryo-TEM) (Talos F200C; Thermo Fisher Scientific, Waltham, Massachusetts, USA).

### In vitro cytotoxicity and lactate dehydrogenase release assays

The in vitro cytotoxicity of CHA-SME was evaluated in C6 and Caco-2 cells using CCK-8 kits (Dojindo Laboratories, Tokyo, Japan). C6 or Caco-2 cells were plated in a 96-well plate at 1×10^4^ cells/well. After 24 hours, the cells were treated with CHA-SME containing various concentrations of CHA (0, 0.01, 0.1, 1, 5, 10, 50, and 100 µM). After incubation for 24 or 48 hours, cell viability was measured using CCK-8 kits according to the manufacturer’s protocol. Untreated cells were used as the control and were considered 100% viable.

The effect of CHA-SME on Caco-2 cell membrane permeability was measured using lactate dehydrogenase (LDH) assay kits (Dojindo Laboratories, Tokyo, Japan). Briefly, Caco-2 cells were plated in a 96-well plate at 1×10^4^ cells/well. After 24 hours, the cells were incubated with CHA-SME containing various concentrations of CHA for 4 hours. After processing per the manufacturer’s protocol, the absorbance at 490 nm was measured. Cells incubated with cell culture medium or lysis buffer were used as the control or positive control, respectively.

### In vitro cellular uptake assay

Cellular uptake of CHA-SME loaded with Cou-6 was qualitatively and quantitatively examined by confocal laser scanning microscopy (CLSM) and flow cytometry, respectively. Briefly, Caco-2 cells were seeded into 12-well plates or glass-bottomed cell culture dishes (NEST Biotechnology, Wuxi, China) at 2×10^5^ cells/well and incubated for 24 hours. Then, the cells were incubated with Cou-6-labeled CHA-SME at 37°C for different periods. For CLSM, the cells were rinsed thrice with cold phosphate-buffered saline (PBS), fixed with 4% paraformaldehyde (PFA), stained with tetramethylrhodamine (TRITC) phalloidin (YEASEN Biotech, Shanghai, China) and 4′,6-diamidino-2-phenylindole (DAPI), and observed by CLSM (TCS SP8X; Leica Microsystems, Wetzlar, Germany). For flow cytometry, the cells were washed thrice with cold PBS, harvested, and analyzed using a flow cytometer (Accuri C6; BD Biosciences, San Jose, CA, USA).

### Endocytosis and exocytosis pathways in Caco-2 cells

To investigate endocytosis pathways, Caco-2 cells were pretreated with transport inhibitors, including chlorpromazine (10 µg/mL), genistein (0.2 mM), amiloride hydrochloride (0.1 mM), and methyl-β-cyclodextrin (M-β-CD) (10 mM), at 37°C for 1 hour.^34 35^ Then, the cells were incubated with Cou-6-labeled CHA-SME containing the transport inhibitors at the same concentrations as described for another 2 hours. After incubation, the Caco-2 cells were washed thrice with cold PBS, harvested, and analyzed by flow cytometry.

To investigate exocytosis pathways, Caco-2 cells were incubated with Cou-6-labeled CHA-SME at 37°C for 2 hours. Then, the cells were washed thrice with PBS and incubated with fresh MEM containing endocellular transport inhibitors, including brefeldin A (25 µg/mL), monensin (32.5 µg/mL), nocodazole (6 µg/mL), and bafilomycin A1 (100 nM), for another 3 hour.^36^ After incubation, intracellular Cou-6 was detected by flow cytometry as described above.

### Transport of CHA-SME within Caco-2 cell monolayers

To establish an in vitro Caco-2 cell monolayer model, Caco-2 cells were plated in a 12-well Transwell plate with a 0.4 µm pore polycarbonate membrane insert (Corning, Corning, New York, USA). A total of 0.5 mL of cell culture medium containing 2×10^5^ cells was added in the upper compartment of each Transwell insert, and 1.5 mL of cell culture medium was added in the lower compartment. The culture medium in both compartments was changed every other day in the initial 14 days and every day in the following 7 days. After 21 days of culture, transepithelial electrical resistance (TEER) was measured using Millicell-ERS (Millipore, Billerica, Massachusetts, USA) to monitor cell monolayer integrity. A TEER value exceeding 800 Ω was selected for subsequent transepithelial transport study of CHA-SME.

For the transepithelial transport study of CHA-SME, Caco-2 cell monolayers cultured in Transwell plates were incubated with Cou-6-labeled CHA-SME for 4 hours at 37°C. Then, the cells were washed thrice with cold PBS, and the polycarbonate membrane with cultured cells was carefully cut from the insert. After fixation with 4% PFA and staining with TRITC phalloidin and DAPI, the excised membrane was placed in a glass slide and examined by CLSM.

### Transport mechanism of CHA-SME across Caco-2 cell monolayers

Caco-2 cell monolayers were incubated with Cou-6-labeled CHA-SME at 37°C for 2 hours. After incubation, the monolayers were washed thrice with Hank’s balanced salt solution (HBSS). Then, fresh HBSS containing the microtubule inhibitor nocodazole was added to both the upper and lower compartments and the monolayers were incubated at 37°C for another 3 hours. Finally, the fluorescence intensity of HBSS on both sides was detected using a multimode microplate reader (Synergy H1; BioTek, Winooski, Vermont, USA). The polycarbonate membrane with cultured cells was carefully cut from the insert and examined by CLSM as described above.

### In vitro CHA-SME uptake in LYM

Cellular uptake of Cou-6-labeled CHA-SME in MLN-derived LYM was qualitatively examined by flow cytometry. Briefly, MLNs were harvested from ICR mice and ground into single-cell suspensions. The LYM obtained were seeded into 12-well plates and incubated with Cou-6-labeled CHA-SME at 37°C for 1 hour. Then, the cells were collected, stained with fluorochrome-labeled anti-mouse monoclonal antibodies, washed thrice with cold PBS, and analyzed by flow cytometry.

To investigate the cellular uptake of Cou-6-labeled CHA-SME in LYM after crossing Caco-2 cell monolayers, LYM were co-cultured with differentiated Caco-2 cell monolayers. After the monolayers achieved optimal and stable TEER values, LYM generated as described above were added basolaterally to the lower compartments. On the addition of LYM, Caco-2 cells were incubated apically with Cou-6-labeled CHA-SME for 4 hours. After incubation, the LYM in the lower compartments were collected, washed thrice with cold PBS, and analyzed by flow cytometry.

### Pharmacokinetic study

Pharmacokinetic studies of free CHA and CHA-SME were performed using SD rats and beagle dogs. Six male SD rats were divided randomly into two groups (CHA and CHA-SME, n=3). A single dose of 30 mg/kg CHA or CHA-SME was orally administered. Beagle dogs (n=3) received a single dose of 30 mg/kg CHA or CHA-SME by oral administration or 3 mg/kg CHA solution via intravenous injection. At 5, 15, 30 min, 1, 2, 4, 6, and 8 hours, approximately 0.2 mL of blood was collected from the orbital into an anticoagulant tube and centrifuged at 4500 rpm for 10 min to separate the plasma. All plasma samples were stored frozen at –80°C until LC-MS/MS analysis (TSQ Quantum Access MAX; Thermo Fisher Scientific, Waltham, MA, USA).

To investigate the lymphatic transport of CHA-SME in vivo, SD rats were intraperitoneally (i.p.) administered 3.0 mg/kg cycloheximide to block chylomicron flow. One hour cycloheximide-injection, CHA-SME was orally administered at a single dose of 30 mg/kg. At 5, 15, 30 min, 1, 2, and 4 hour, approximately 0.2 mL of blood was collected and processed as described above.

### Biodistribution of CHA-SME in the intestinal tracts and MLNs

A subcutaneous glioma tumor model was established by subcutaneous injection of 2×10^6^ G422 cells into the right flank of ICR mice. When the tumors reached a median size of 500 mm^3^, DiR solution and DiR-labeled CHA-SME were orally administered at 1.0 mg/kg. At 0.5, 1, 2, and 4 hours, the mice were sacrificed and the intestinal tracts and MLNs were surgically collected. Fluorescence images of the intestinal tracts and MLNs were taken using an In Vivo IVIS spectrum-imaging system (PerkinElmer, Waltham, Massachusetts, USA).

To evaluate the uptake of CHA-SME in the intestinal tissues and MLNs, Cou-6-labeled CHA-SME were orally administered to mice at 1 mg/kg. Thirty minutes later, the mice were sacrificed and intestinal segments (including duodenum, jejunum, ileum, and colon,~1 cm each) and MLNs were surgically collected, placed in embedding compound, frozen in liquid nitrogen, and dissected. The slices were fixed with 4% PFA, stained with TRITC phalloidin and DAPI, and imaged using CLSM. Additionally, MLNs were ground into single-cell suspensions and the fluorescence intensity was determined by flow cytometry.

To investigate the lymphatic transport of CHA-SME in vivo, mice were i.p. administered 3.0 mg/kg cycloheximide to block chylomicron flow.^37 38^ One-hour postinjection, DiR-labeled or Cou-6-labeled CHA-SME was orally administered at 1.0 mg/kg. The mice were examined as described above.

### In vivo antitumor efficacy

An orthotopic glioma model was established by implantation of G422 cells in the brains of ICR mice. Briefly, female ICR mice were anesthetized with pentobarbital sodium and placed in a stereotaxic restraint. G422 cells (2×10^6^) were implanted into the right brain at a speed of 10 µL/min. Tumor-bearing mice were randomly divided into six groups on the day after tumor implantation. The positive control group was orally administered chemotherapeutic agent TMZ (50 mg/kg) for 5 days. The CHA group was intraperitoneally injected a CHA solution (10 and 20 mg/kg) for 9 days. The CHA-SME group was orally administered CHA-SME (20 and 35 mg/kg) for 9 days. At the end of the experiment, the brains were imaged by (magnetic resonance imaging) MRI (PharmaScan 70/16 US; Bruker, Karlsruhe, Baden-Württemberg, Germany). Fresh tumors were collected and analyzed to evaluate the in vivo immunoregulatory effect.

A subcutaneous glioma tumor model was established in female ICR mice, as mentioned above. To investigate whether blank CHA-SME had an effect on glioma tumor growth, the model mice were randomly divided into three groups (control, positive control, and blank CHA-SME, n=6 per group) the day after tumor implantation. The positive control group was orally administered TMZ (50 mg/kg) for 5 days. The blank CHA-SME group was orally administered with blank CHA-SME for 14 days. At the end of the experiment, all mice were euthanized, and the tumors were collected, weighed, and photographed. To compare the antitumor effects of CHA in different formulations, tumor-bearing mice were randomly divided into four groups (n=7 per group) the day after tumor implantation. The positive control group was orally administered TMZ (50 mg/kg) for 5 days. The CHA group was i.p. injected a CHA solution (20 mg/kg) for 14 days. The CHA-SME group was orally administered CHA-SME (35 mg/kg) for 14 days. At the end of the experiment, all mice were euthanized, and the tumors were collected, weighed, and photographed. The tumor growth inhibition ratio (TGI%) was calculated using the following formula: TGI% = (1 – W_test_/W_control_)×100%, where W_test_ and W_control_ represent mean tumor weight in the treatment and control groups, respectively.

### Immunomodulatory effects of CHA-SME in vivo

The in vivo immunomodulatory effects of CHA-SME were evaluated by analyzing the immunophenotype of LYM and cytokine production. LYM (DCs and T cells) infiltrated into the blood, tumor, and other important immune organs (MLNs and spleen) were quantitatively analyzed by flow cytometry. Fresh tumors were cut into small pieces and digested at 37°C for 40 min. Fresh MLNs and spleens were cut into small pieces and ground into single-cell suspensions. The cells were collected, washed, and incubated with different fluorescein-conjugated antibodies. The cell suspensions were filtered through 400-mesh sieves after two washes and analyzed by flow cytometry (NovoCyte D3010; AECA Biosciences, San Diego, California, USA). For immunophenotype analysis of T cells, CD45 +CD3+CD44+CD62L+cells and CD45 +CD3+CD44+CD62 L-cells were considered to be central memory T cells (T_CM_) and effector memory T cells (T_EM_), respectively. For DC maturation analysis, the expression levels of MHC I, CD40, CD80, and CD86 on DCs were examined. Different cytokines (IFN-γ, tumor necrosis factor α (TNF-α), and IL-10) in tumors, MLNs, spleens, and blood were quantitatively determined using bead-based LEGENDplex kits following the manufacturer’s protocol.

To investigate whether CHA-SME treatment increases the risk of inducing the cytokine storm in vivo, normal mice were randomly divided into three groups (control, positive control, and CHA-SME, n=7 per group). The positive control group was administered with a single i.p. injection of LPS (5 mg/kg). The CHA-SME group was orally administered with CHA-SME (35 mg/kg) for consecutive 14 days. At the end of the experiment, peripheral blood was collected and cytokines were quantitatively determined using bead-based LEGENDplex kits following the manufacturer’s protocol.

### In vivo safety evaluation

The in vivo safety of CHA-SME was evaluated based on body weight, hematological examination, and histopathological examination in mice. Body weight was monitored throughout the experiment. At the end of the in vivo antitumor experiment, peripheral blood was collected for hematological examination using an automatic hematology analyzer (MEK-7222 K; Nihon Kohden, Tokyo, Japan). Additionally, the serum was separated to detect alkaline aminotransferase (ALT), aspartate phosphatase (AST), blood urea nitrogen (BUN), and creatinine (CRE) to evaluate hepatic and renal functions using an automatic biochemical analyzer (Accute TBA-40FR; TOSHIBA, Kawasaki, Japan). Heart, liver, spleen, lung, kidney, stomach, small intestine, and MLNs were collected, fixed in 4% PFA, and subjected to histopathological examination using HE staining.

### Statistical analysis

All data subjected to statistical analysis were obtained from at least three parallel experiments. The statistical analysis was performed by unpaired two-tailed Student’s t-test for two groups, and one-way analysis of variance for multiple groups using GraphPad Prism V.7.00 for Windows (GraphPad Software, La Jolla, California, USA). A p≤0.05 was considered statistically signiﬁcant.

## Results and discussion

### Properties and characteristics of CHA-SME

SMEDDS consist of an oil, a surfactant, a cosurfactant, and the active pharmaceutical ingredient, and should spontaneously and quickly form a clear and monophasic microemulsion at room temperature when introduced into an aqueous phase. In addition to the easy preparation process, SMEDDS hold great potential for MLNs-targeted delivery owing to their ability to improve drug solubilization and membrane permeability in the gastrointestinal tract and promote the drug access to LYM concentrated within in the MLNs via intestinal lymphatic transport.^39^ We prepared SMEDDS using ethyl oleate, Labrasol, and Transcutol HP as the oil, surfactant, and co-surfactant, respectively. Labrasol is a widely used medium-length alkyl chain surfactant with outstanding biological characteristics, especially the ability to transiently induce mild intestinal epithelial perturbation and reversibly open intestinal epithelial tight junctions to promote intestinal drug transport.^40^ CHA is a promising candidate for cancer immunotherapy because of its immunomodulatory activities on key immune cells, such as tumor-associated macrophages, DCs, and T cells.^19 20 23 24^ However, as a hydrophilic small-molecule compound, poor lipophilicity of CHA impedes its association with the oily droplet core of SMEDDS and its permeation into the intestinal tract. A sufficient degree of lipophilicity of a therapeutic agent is crucial for successful incorporation into the oily droplet core of SMEDDS.^41^ Phospholipids have a long fatty acid chain (lipophilic part); therefore, the lipophilicity of CHA can be improved by the addition of phospholipids to form CHA-phospholipid complexes.^23 24^ In the present study, we used phospholipid complexes to increase the lipophilicity of CHA, and SMEDDS loaded with CHA-phospholipid complexes were successfully developed and evaluated for the promotion of intestinal lymphatic transport, the subsequent immunomodulatory effect, and cancer immunotherapeutic efficacy in vitro and in vivo.

**Figure 2:**
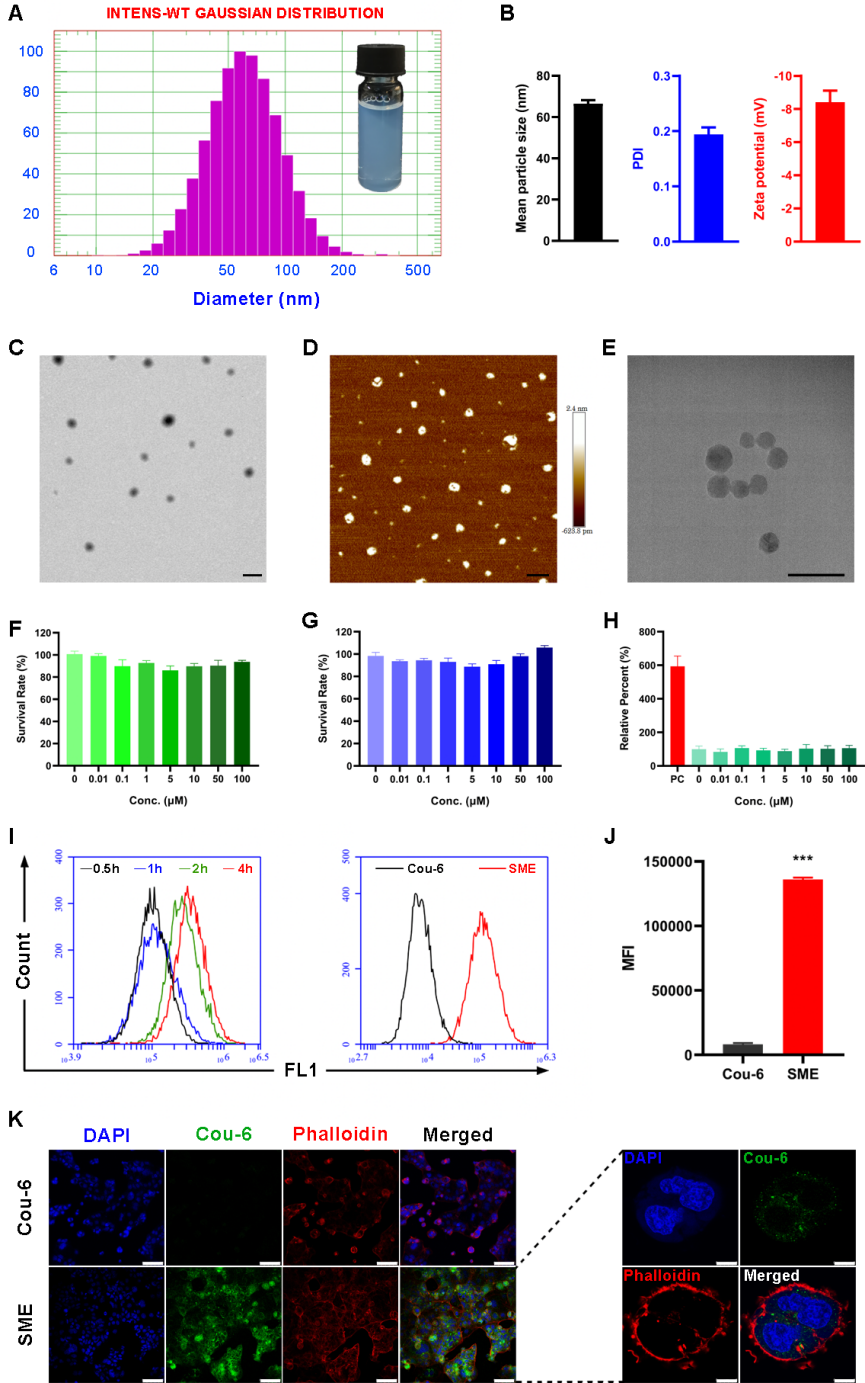
In vitro characterization, in vitro cytotoxicity, and cellular uptake profile of CHA-SME. (A) Particle size distribution and appearance of CHA-SME. (B) Mean particle size, PDI, and zeta potential of CHA-SME. (C–E) TEM, AFM, and cryo-TEM images of CHA-SME. Scale bar=200 nm. (F, G) Cytotoxicity of CHA-SME in Caco-2 and C6 glioma cells determined by CCK-8 kits. Each value represents the mean±SD (n=4–6). (H) The impact of CHA-SME on Caco-2 cell membrane integrity evaluated by the LDH release assay. PC represents positive control. Each value represents the mean±SD (n=6). (I, J) The in vitro cellular uptake profile of Cou-6-labeled CHA-SME in Caco-2 cells determined by flow cytometry. Each value represents the mean±SD (n=3). ***P<0.001 compared with the Cou-6. (K) CLSM images of in vitro cellular uptake profile of Cou-6-labeled CHA-SME in Caco-2 cells. The cell nuclei and cytoskeleton were stained with DAPI (blue) and phalloidin (red), respectively. Scale bar=100 µm (left), scale bar=8 µm (right). AFM, atomic force microscopy; CHA-SME, chlorogenic acid-encapsulated SMEDDS; CLSM, confocal laser scanning microscopy; LDH, lactate dehydrogenase; MFI, mean fluorescence intensity; PDI, polydispersity Index; TEM, transmission electron microscopy.

The efficiency of self-emulsification is a pivotal parameter to evaluate the spontaneity of SMEDDS emulsification and can be estimated by determining the emulsification time and droplet size. The emulsification time of CHA-SME was within 30 s when diluted in water, indicating that the formulation could rapidly self-microemulsify. After self-microemulsification in distilled water, the microemulsion was slightly blue opalescent under light. The mean particle size and zeta potential of the microemulsion were 66.5±1.3 nm and –8.4±0.6 mV, respectively (figure 2A, B). The low Polydispersity Index values (~0.2) demonstrated a uniform size distribution of the microemulsion droplets. TEM, AFM, and cryo-TEM images of the microemulsion revealed nano-sized globules of similar size (60–100 nm) (figure 2C–E), which was consistent with the DLS result.

The droplet size of microemulsions has a significant impact on the rate and extent of drug release as well as on intestinal absorption and bioavailability of the therapeutic agent. A smaller droplet size can positively affect the intestinal absorption of SMEDDS.^10^ Importantly, access to the lymphatics is determined by the size of nanoparticles. Nanoparticles of 10–100 nm diameters are preferentially taken up via lymphatic vessels, whereas larger nanoparticles (>100 nm) are trapped in the interstitial matrix.^1 2^ Apart from the droplet size, the surface charge of nanocarriers affects lymphatic transport in the following order: negatively charged >positively charged >neutral.^11^ Thus, the ~60 nm size and negative zeta potential of CHA-SME will promote access to the MLNs.

### Effect of CHA-SME on proliferation and membrane integrity of Caco-2 cells

We previously reported that CHA acts as an antitumor immunomodulator inhibiting glioblastoma growth by promoting the polarization of tumor-associated macrophages from M2 to M1 phenotype rather than directly exerting toxicity on tumor cells.^20 23^ Although CHA inhibits cell proliferation and induced cell apoptosis in several human cancer cell lines, the effective dose of CHA is higher than 100 µM or the maximum dose of 500 µM.^42–44^ To investigate the direct cytotoxicity of both CHA and CHA-SME to tumor cells, the proliferation and viability of C6 glioma and Caco-2 cells treated with CHA-SME were evaluated using CCK-8 kits. The survival rate after incubation with CHA-SME at CHA concentrations of 0.01–100 µM was near 100% (figure 2F, G), indicating significant biocompatibility of CHA-SME with Caco-2 and C6 glioma cells and negligible cytotoxicity of CHA at 100 µM to these cells.

The effect of CHA-SME on cell membrane integrity was evaluated using the LDH release assay. LDH release indicates a change in cell membrane permeability and reflects the extent of damage.^45^ LDH release from CHA-SME with various concentrations of CHA (0.01–100 µM) was comparable to that in the control group (figure 2H), indicating a negligible impact of CHA-SME on cell membrane integrity. The negligible effect of the CHA-SME on proliferation and membrane integrity of Caco-2 cells was the foundation of the following studies of transport mechanisms. Based on the results presented, we speculate that the endocytosis of CHA-SME by Caco-2 cells and transport of CHA-SME across Caco-2 cell monolayers in the following experiments cannot be attributable to the cell membrane damage.

### In vitro cellular uptake profile of CHA-SME

As shown in figure 2I–K, Caco-2 cells treated with Cou-6-labeled CHA-SME exhibited a significantly higher mean fluorescence intensity (MFI) than those treated with free Cou-6 solution, demonstrating that CHA-SME significantly facilitated cellular uptake of the encapsulated agent. Additionally, CHA-SME uptake increased significantly with prolonged incubation time, indicating that the internalization process of CHA-SME in Caco-2 cells is time-dependent.

### Endocytosis and exocytosis pathways of CHA-SME in Caco-2 cells

To better understand the cellular internalization mechanism of CHA-SME, the speciﬁc endocytosis inhibitors chlorpromazine, genistein, amiloride hydrochloride, and M-β-CD were used to inhibit clathrin-dependent, caveolae-dependent, macropinocytosis-dependent, and lipid raft/caveolae-dependent endocytosis, respectively.^36^ As shown in online supplemental figure S1A, CHA-SME uptake in Caco-2 cells signiﬁcantly decreased when lipid raft/caveolae-dependent endocytosis was inhibited by selective cholesterol extraction with M-β-CD. The addition of free cholesterol partially counteracted M-β-CD-mediated endocytosis inhibition (online supplemental figure S1B), confirming that CHA-SME endocytosis in Caco-2 cells involved the lipid raft/caveolae pathway.^46^ Amiloride hydrochloride had no effect on CHA-SME uptake. Compared with the control, both chlorpromazine and genistein enhanced rather than reduced the cellular uptake of CHA-SME. Increased cellular uptake of agents after pretreatment with speciﬁc endocytosis inhibitors has been previously reported.^47 48^ It is speculated that additional endocytosis pathways that are not normally involved may be activated in the presence of inhibitors.^35 49^ In summary, these results demonstrated that CHA-SME internalization into Caco-2 cells primarily relies on lipid raft/caveolae-dependent endocytosis pathways.

10.1136/jitc-2021-002753.supp1 Supplementary data

After internalization in the cytoplasm or organelles, CHA-SME will be exported from Caco-2 cells. As the reverse trafficking process of endocytosis, exocytosis here specifically refers to the exocytosis of internalized CHA-SME from the apical plasma membrane of Caco-2 cells. To investigate the exocytosis mechanism of CHA-SME, the transepithelial transport amount of Cou-6-labeled CHA-SME was measured in the presence of the endocellular transport inhibitors brefeldin A, monensin, nocodazole, and bafilomycin A1 (online supplemental figure S1C).^14 36 46^ Brefeldin A, which triggers the retrograde transport of Golgi enzymes back to the endoplasmic reticulum (ER), inhibits the ER/Golgi apparatus pathway. Monensin blocks the transport of macromolecules from the Golgi apparatus to the plasma membrane by disrupting the Golgi complex function. Compared with the control group, brefeldin A and monensin significantly enhanced CHA-SME accumulation in Caco-2 cells, indicating that both ER/Golgi apparatus and Golgi apparatus/plasma membrane pathways play an important role in CHA-SME exocytosis. Nocodazole inhibits vesicles transport to the cell plasma membrane by specifically blocking the microtubules. Treatment with nocodazole resulted in a significant enhancement of CHA-SME accumulation, demonstrating that microtubules are principally involved in the exocytosis of CHA-SME. Bafilomycin A1, which inhibits the maturation of early endosomes to lysosomes, was used to evaluate the role of this maturation process in the exocytosis of CHA-SME. After treatment with bafilomycin A1, there was no obvious change in the cellular accumulation of CHA-SME in comparison with the control group, indicating that the maturation process of early endosomes to lysosomes had no effect on the exocytosis of CHA-SME. These results suggested that the exocytosis process of CHA-SME is complex and mediated by multiple pathways. The ER, Golgi apparatus, and microtubules have critical regulatory functions in the export of CHA-SME from Caco-2 cells.

### Transport of CHA-SME within Caco-2 cell monolayers

**Figure 3:**
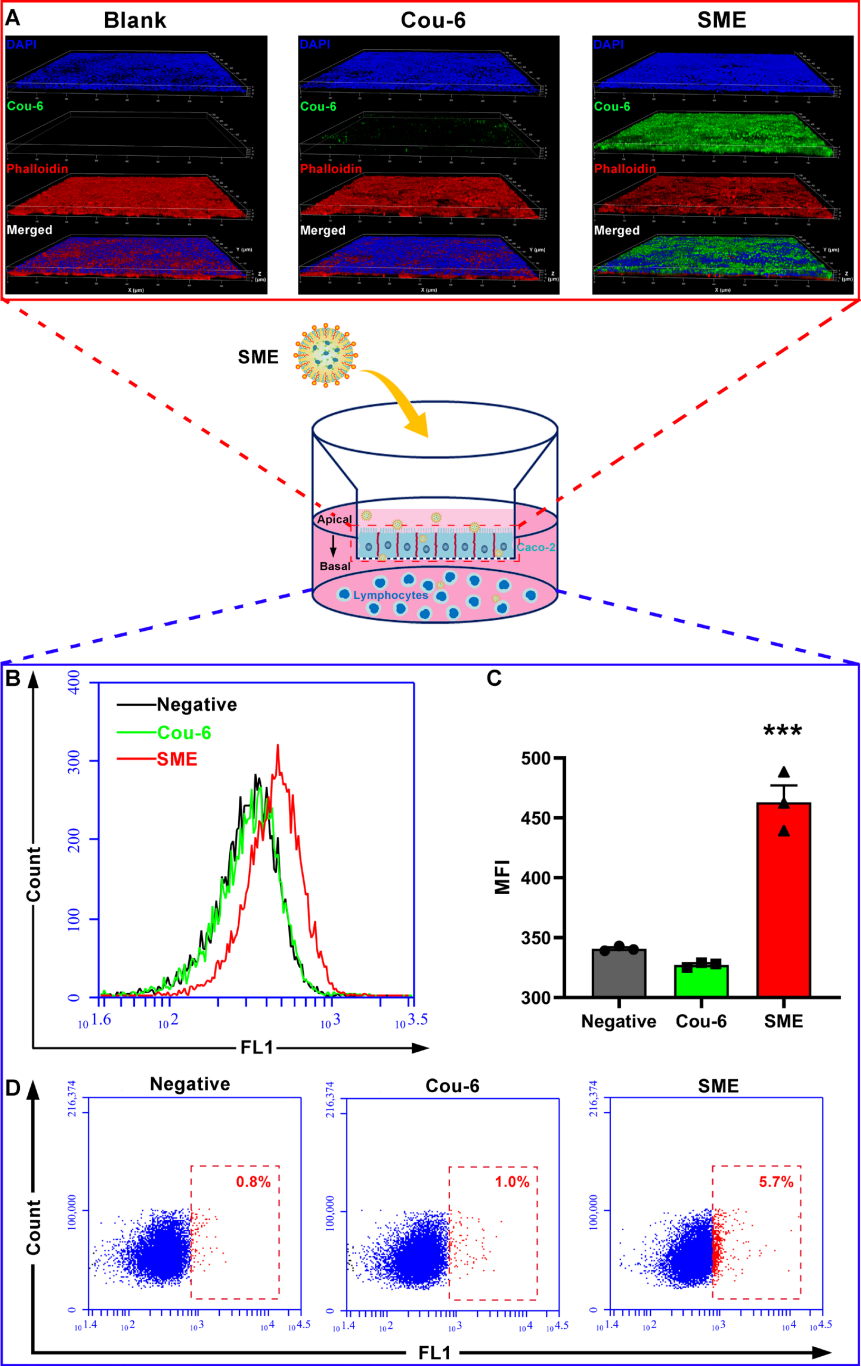
Transport profile of CHA-SME within Caco-2 cell monolayers. (A) CLSM images of Caco-2 cell monolayer after incubation with Cou-6-labeled CHA-SME. the cell nuclei and cytoskeleton were stained with DAPI (blue) and phalloidin (red), respectively. (B, C) The in vitro cellular uptake profile of Cou-6-labeled CHA-SME in lymphocytes determined by flow cytometry. Each value represents the mean±SEM (n=3). ***P<0.001 compared with the Cou-6. (D) The percentage of Cou-6-positive lymphocytes determined by flow cytometry. CHA-SME, chlorogenic acid-encapsulated SMEDDS; CLSM, confocal laser scanning microscopy.

The effectiveness of CHA-SME in promoting the transcellular delivery of encapsulated agent across Caco-2 cell monolayers was evaluated qualitatively and quantitatively using CLSM and flow cytometry, respectively. As shown in figure 3A, Caco-2 cell monolayers administered free Cou-6 exhibited negligible green fluorescence, whereas significantly higher green fluorescence was observed in the Cou-6-labeled CHA-SME group. This finding, consistent with the in vitro cellular uptake assay results, indicated that CHA-SME significantly promotes the cellular accumulation of encapsulated agents within Caco-2 cell monolayers, which may contribute to facilitating transcellular delivery across cell monolayers.

The enterocytes in the intestinal tract are surrounded by the lamina propria and are connected with abundant blood capillaries and lymphatic vessels. Once a therapeutic agent is transported across the intestinal epithelium, it can drain into the MLNs through the lymphatic vessels.^39^ To investigate whether CHA-SME can promote transcellular delivery across cell monolayers and can be taken up by LYM in the MLNs after crossing the intestinal barrier, a coculture model of LYM and Caco-2 cell monolayers was established. As shown in figure 3B–D, the CHA-SME-treated group exhibited a significantly higher MFI and percentage of Cou-6-positive LYM than the free Cou-6-treated group, indicating that CHA-SME can not only cross Caco-2 cell monolayers but also is efficiently taken up by LYM soon afterward. Additionally, the cellular uptake index of CHA-SME was significantly higher than that of free Cou-6 when each was directly incubated with LYM prepared from MLNs (online supplemental figure S2). As shown in online supplemental figure S3, the MFI in CD11c+DCs was higher than that in CD3 +T cells. It is generally considered that CHA-SME does not have any special subset of LYM-targeting properties as the surface of CHA-SME is not decorated with the cell-specific targeting ligand. The higher cellular uptake index of CHA-SME in DCs may be attributed to the phagocytic activity of DCs.

### Transport mechanism of CHA-SME across Caco-2 cell monolayers

CHA-SME are endocytosed within the epithelial cells from the apical membrane and then, the internalized particles are discharged via the apical or basolateral membrane. To investigate the transport mechanism related to the exocytosis process of the CHA-SME from Caco-2 cell monolayers, the microtubule inhibitor nocodazole, which significantly hinders the exocytosis process of CHA-SME from Caco-2 cells (online supplemental figure S1C), was used to investigate the discharge of the CHA-SME via both membrane sides. Consistent with the exocytosis of the CHA-SME from Caco-2 cells, microtubules disruption significantly reduced the transcytosis of CHA-SME in Caco-2 cell monolayers (online supplemental figure S4A). As nocodazole significantly inhibited the transcytosis of the CHA-SME, the higher green fluorescence was accumulated within Caco-2 cell monolayers as compared with the control group (online supplemental figure S4B, C), confirming that microtubules play an important role in the transport of CHA-SME across Caco-2 cell monolayers.

### Pharmacokinetic study

**Figure 4:**
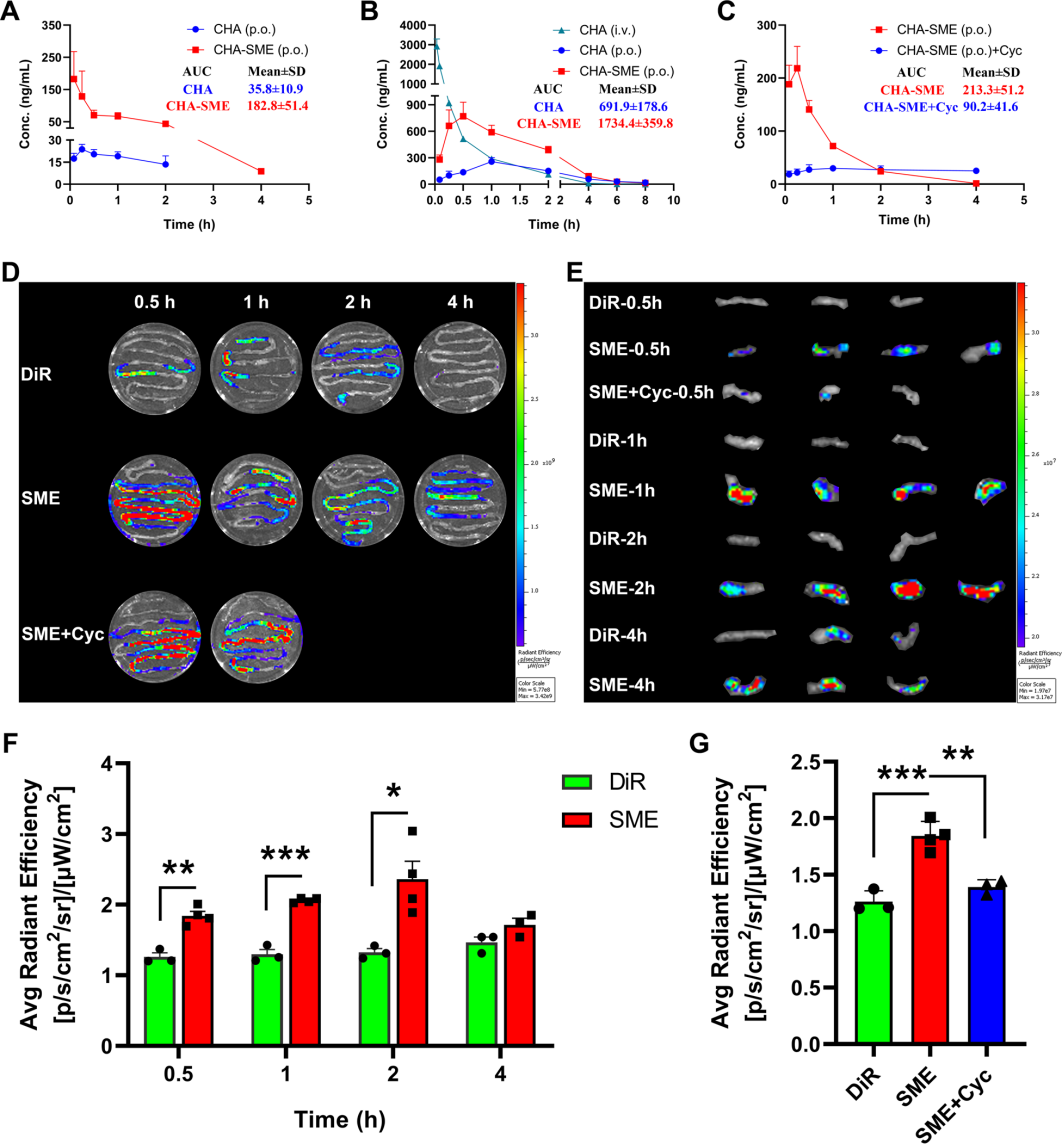
In vivo pharmacokinetic profile and biodistribution. (A, B) Mean plasma concentration-time curves of CHA after oral administration to rats and beagle dogs, respectively. p.o., orally; i.v., intravenously. Each value represents the mean±SEM (n=3). (C) Mean plasma concentration-time curves of Cha from CHA-SME after oral administration to rats pretreated with or without cycloheximide. ‘CHA-SME (p.o.)+Cyc’ represents the rats that are pretreated with cycloheximide and orally administered CHA-SME. Each value represents the mean±SEM (n=5). (D) Ex vivo imaging of the distribution of DiR-labeled CHA-SME in intestinal tract after oral administration at predetermined time points. ‘SME+Cyc’ represents the mice that are pretreated with cycloheximide and orally administered with DiR-labeled CHA-SME. The same as below. (E) Ex vivo imaging of the distribution of DiR-labeled CHA-SME in MLNs after oral administration at predetermined time points. (F) The quality of DIR and DiR-labeled CHA-SME accumulated in MLNs at predetermined time points. each value represents the mean±SEM (n=3–4). *P<0.05, **p<0.01, ***p<0.001. (G) The quality of DiR and DiR-labeled CHA-SME accumulated in MLNs at 0.5 hour. Each value represents the mean±SEM (n=3–4). **P<0.01, ***p<0.001. CHA-SME, chlorogenic acid-encapsulated SMEDDS; DiR, 1,1-dioctadecyl-3,3,3,3-tetramethylindotricarbocyanine iodide; MLNs, mesenteric lymph nodes.

The mean plasma concentration-time profiles of free CHA and CHA-SME in rats and beagle dogs are shown in figure 4A, B. The plasma CHA concentration was significantly higher in the CHA-SME group than in the free CHA group at all time points after oral administration. In rats, the AUC and C_max_ of CHA-SME were 5.1-fold and 9.5-fold higher than those of free CHA, respectively. In beagle dogs, the AUC and C_max_ of CHA-SME were 2.5- and 2.9-fold higher than those of free CHA, respectively. The oral absolute bioavailability of CHA-SME (14.86%±4.02%) was 2.5-fold higher than that of CHA (5.85%±1.4%). These results indicated that CHA-SME effectively facilitates the oral absorption of CHA in vivo potentially by improving drug permeability in the intestine.

Following transport across the intestinal epithelium, a therapeutic agent can enter into the blood circulation via two pathways: the portal blood and lymphatic transport.^39^ Thus, portal blood is the only absorption pathway when lymphatic transport is blocked. To investigate the fraction of drug transported by the intestinal lymphatic system, we measured the plasma CHA concentration following oral administration of CHA-SME to rats pretreated with saline or cycloheximide. The fraction transported by the lymphatic pathway was calculated by subtracting the fraction transported to the blood circulation in rats pretreated with cycloheximide from the total bioavailability in rats pretreated with saline.^10 37^ As shown in figure 4C, in cycloheximide pretreated rats, C_max_ and AUC of CHA were reduced by 81.9% and 57.7%, respectively, indicating that the intestinal lymphatic transport is a major pathway of CHA-SME absorption.

### Biodistribution of CHA-SME in the intestinal tract and MLNs

The in vivo oral biodistribution of DiR-labeled CHA-SME in the intestinal tract and MLNs was determined by fluorescence imaging in murine G422 glioma tumor-bearing mice. As shown in figure 4D, ex vivo fluorescence images of intestinal tracts revealed that the DiR-SME-treated group presented a significantly higher fluorescence intensity than the DiR-DMSO-treated group at all time points observed (0.5, 1, 2, and 4 hours) following oral administration. More notably, the fluorescence intensity in MLNs from DiR-SME-treated mice was higher than that in MLNs from DiR-DMSO-treated mice at all time points observed (figure 4E, F). These results demonstrated that CHA-SME promote the accumulation of the encapsulated agent in the intestinal tract and then efficiently target the MLNs.

To investigate whether the increased accumulation within the MLNs is mediated by lymphatic transport, mice were i.p. administered cycloheximide to block chylomicron flow and were then orally administered DiR-labeled CHA-SME. As shown in figure 4D, the fluorescence intensity in the intestinal tract was significantly higher in the group pretreated with cycloheximide (DiR-SME +Cyc) than in the control group (DiR-SME). The enhanced fluorescence accumulation in the small intestine may be attributed to the inhibition of lymphatic transport by cycloheximide because of which CHA-SME cannot be absorbed into the blood via the lymphatic transport pathway through MLNs. As expected, pretreatment with cycloheximide significantly reduced the fluorescence accumulation of CHA-SME in the MLNs (figure 4E, G). These results indicated that CHA-SME effectively facilitate the accumulation of an encapsulated agent within the MLNs via the lymphatic transport pathway.

**Figure 5:**
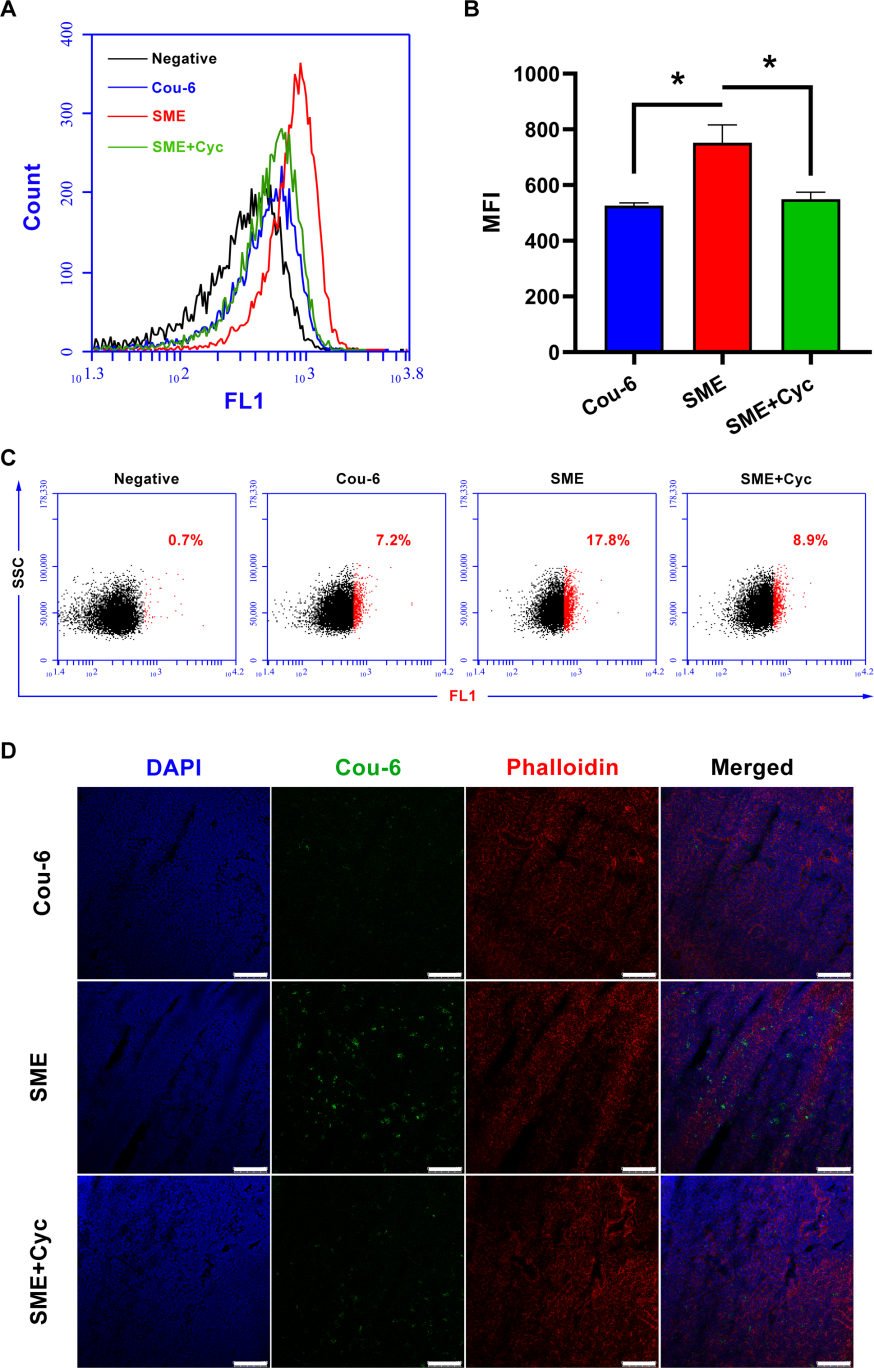
The accumulation of Cou-6-labeled CHA-SME in MLNs after oral administration. (A, B) The MFI of Cou-6 labeled CHA-SME accumulated in MLNs determined by flow cytometry. Each value represents the mean±SEM (n=3). *P<0.05. (C) The percentage of Cou-6-positive lymphocytes determined by flow cytometry. (D) CLSM images of MLNs frozen tissue sections. Scale bar=100 µm. The cell nuclei and cytoskeleton were stained with DAPI (blue) and phalloidin (red), respectively. ‘SME’ represents the mice that are orally administered Cou-6-labeled CHA-SME. ‘SME +Cyc’ represents the mice that are pretreated with cycloheximide and orally administered Cou-6-labeled CHA-SME. CHA-SME, chlorogenic acid-encapsulated SMEDDS; CLSM, confocal laser scanning microscopy; MFI, mean fluorescence intensity; MLNs, mesenteric lymph nodes.

To further confirm the enhanced CHA-SME absorption in the intestinal tract and increased accumulation within the MLNs, the fluorescence distribution of orally administered Cou-6-labeled CHA-SME in the intestinal tract and MLNs was evaluated by CLSM. Consistent with the ex vivo fluorescence imaging results, mice given Cou-6-labeled CHA-SME exhibited a higher fluorescence intensity in the MLNs and duodenum than those given free Cou-6 (figure 5D and online supplemental figure S5A). After pretreatment with cycloheximide, the green fluorescence intensity in the duodenum and MLNs increased and decreased, respectively, which may be attributed to the inhibition of lymphatic transport by cycloheximide. Quantification of the MFI (figure 5A, B)and the percentage of Cou-6-positive LYM (figure 5C) corroborated enhanced accumulation of CHA-SME within the MLNs via the lymphatic transport pathway. Additionally, Cou-6-labeled CHA-SME was distributed mainly in the duodenum, and to a lesser extent in the jejunum and ileum (online supplemental figure S5C, D). These results further confirmed that CHA-SME can enhance drug permeation through the intestinal epithelial cells and promote drug accumulation within the MLNs via the lymphatic transport. Thus, CHA-SME hold potential as an effective MLNs-targeting carrier to deliver immunomodulators to the MLNs.

### In vivo antitumor efficacy

**Figure 6:**
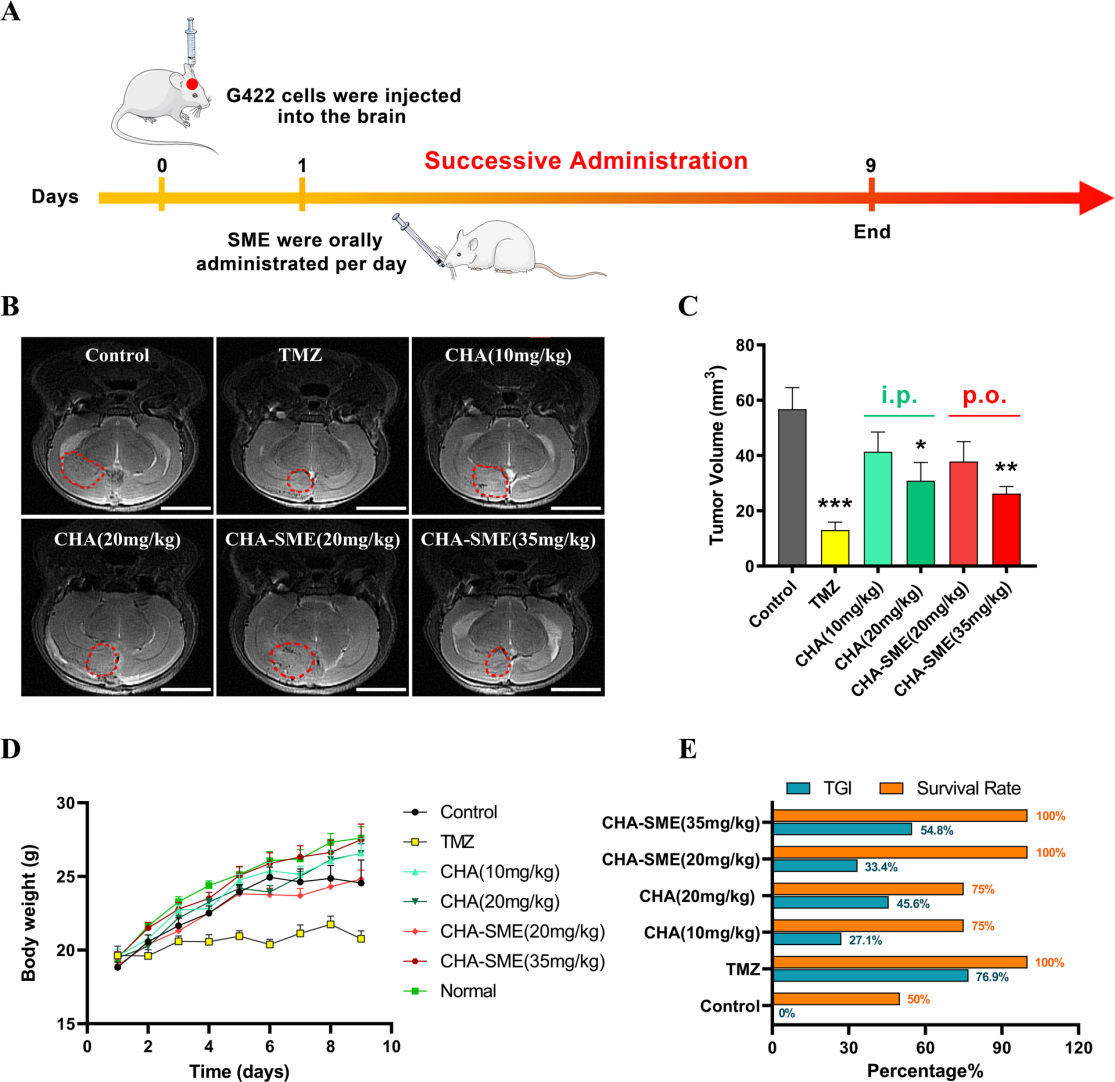
In vivo antitumor efficacy of CHA-SME in an orthotropic G422 glioma murine model. (A) The schematic of treatment schedule. Representative MRI images of intracranial tumors (B), tumor volume (C), body weight changes (D), and tumor growth inhibition ratio (TGI) and survival rate (E) of orthotropic murine G422 glioma tumor-bearing mice after treatment of CHA-SME. Red dotted circles indicate the tumor regions. Scale bar=4 mm. Each value represents the mean±SEM (n=5–8). *P<0.05, **p<0.01 and ***p<0.001 compared with the control group. CHA-SME, chlorogenic acid-encapsulated SMEDDS; i.p., intraperitoneally; p.o., orally; TMZ, temozolomide.

To investigate the antitumor efficacy of CHA-SME against glioma, an orthotopic G422 glioma model mice were treated with CHA-SME, and tumor growth was monitored by MRI. The schedule of administration is illustrated in figure 6A. As shown in figure 6B, C, CHA was able to inhibit the glioma growth in a dose-dependent manner after i.p. injection of CHA solution for nine consecutive days. CHA-SME (35 mg/kg, p.o.) exhibited better antitumor efficacy than free CHA (20 mg/kg, i.p.), as evidenced by the smaller tumor volume, the higher TGI%, and the higher survival rate (figure 6C, E), corroborating the therapeutic potential of CHA-SME against glioma.

The in vivo antitumor efficacy of CHA-SME against glioma was also evaluated in a subcutaneous G422 glioma murine model. The schedule of administration is illustrated in online supplemental figure S6A. As expected, blank CHA-SME did not yield antitumor effects against G422 glioma (online supplemental figure S6B, C). As shown in online supplemental figure S6D, E, both free CHA and CHA-SME were able to inhibit tumor growth after successive administration for 14 days. Consistent with the results in the orthotopic G422 glioma model, CHA-SME exhibited the greater antitumor activity at a dose of 35 mg/kg, as evidenced by the lower tumor weight and higher TGI% as compared with the CHA group (online supplemental figure S6F). Notably, the in vivo antitumor efficacy of CHA-SME was comparable to that of TMZ, a first-line drug for glioma in clinic. The superior antitumor efficacy of CHA-SME than that of CHA in the subcutaneous and orthotopic G422 glioma model may be attributed to the enhanced CHA-SME accumulation within the MLNs via the lymphatic transport, the sustained CHA release, and immune cells activation.

### In vivo immunomodulatory effects

An effective antitumor immune response requires stimulation of DC maturation, T-cell activation and proliferation, and maintenance of the T-cell response long enough for the T cells to effectively eliminate cancer.^50^ The MLNs, which accommodate more than half of the body’s LYM, including T cells and DCs, is a major site for antigen presentation and immune cell activation and have been identified as a target site for immunotherapy.^51^ As a potential immunotherapeutic agent, CHA has a certain effect on T-cell activation and proliferation, which are critical for the outcome of T cell-based immunotherapy.^19 24^ Therefore, targeted delivery of CHA to the MLNs by CHA-SME has a distinct advantage in efficiently regulating the immunomodulatory functions of DCs and T cells in the MLNs. Encouraged by the potency of CHA-SME to inhibit tumor growth in both subcutaneous and orthotopic glioma models, we examined the in vivo immunomodulatory effects of CHA-SME on DCs and T cells.

**Figure 7:**
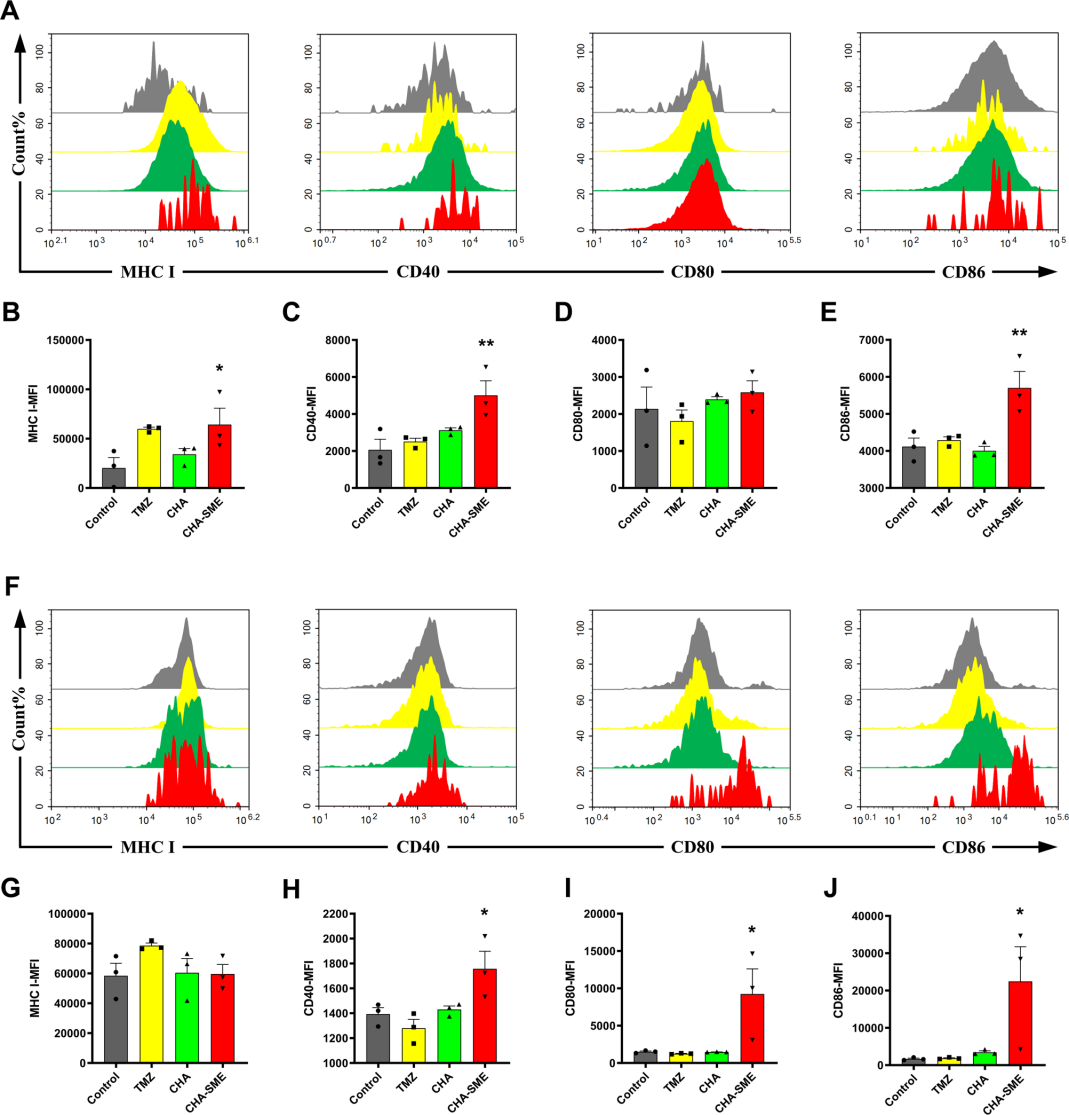
The effects of CHA-SME on DC maturation. (A–E) Flow cytometer analysis for the expression of DC maturation markers: MHC I, CD40, CD80, and CD86 of DCs in MLNs. (F–J) Flow cytometer analysis for the expression of DC maturation markers: MHC I, CD40, CD80, and CD86 of DCs in peripheral blood. Each value represents the mean±SEM (n=3). *P<0.05, **p<0.01 compared with the control group. CHA-SME, chlorogenic acid-encapsulated SMEDDS; DC, dendritic cell; MHC, major histocompatibility complex; MLNs, mesenteric lymph nodes; TMZ, temozolomide.

DCs are the most potent antigen-presenting cells that play a key role in directing T cell-mediated immunotherapy. Antigen presentation by mature DCs is a prerequisite for the activation and proliferation of T cells. To explore the effects of CHA-SME on DC maturation, the expression of classical DC maturation markers, including MHC I and the costimulatory molecules CD40, CD80, and CD86, was quantified by flow cytometry.^52 53^ As shown in figure 7A–E, expression levels of MHC I, CD40, and CD86 in MLNs of the CHA-SME-treated group were signiﬁcantly higher than those in MLNs of other treatment groups, indicating that CHA-SME effectively stimulates resident DC maturation in the MLNs. In addition, CHA-SME induced a pronounced upregulation of CD40, CD80, and CD86 expression in peripheral blood (figure 7F–J), confirming that CHA-SME effectively promotes DC maturation.

**Figure 8:**
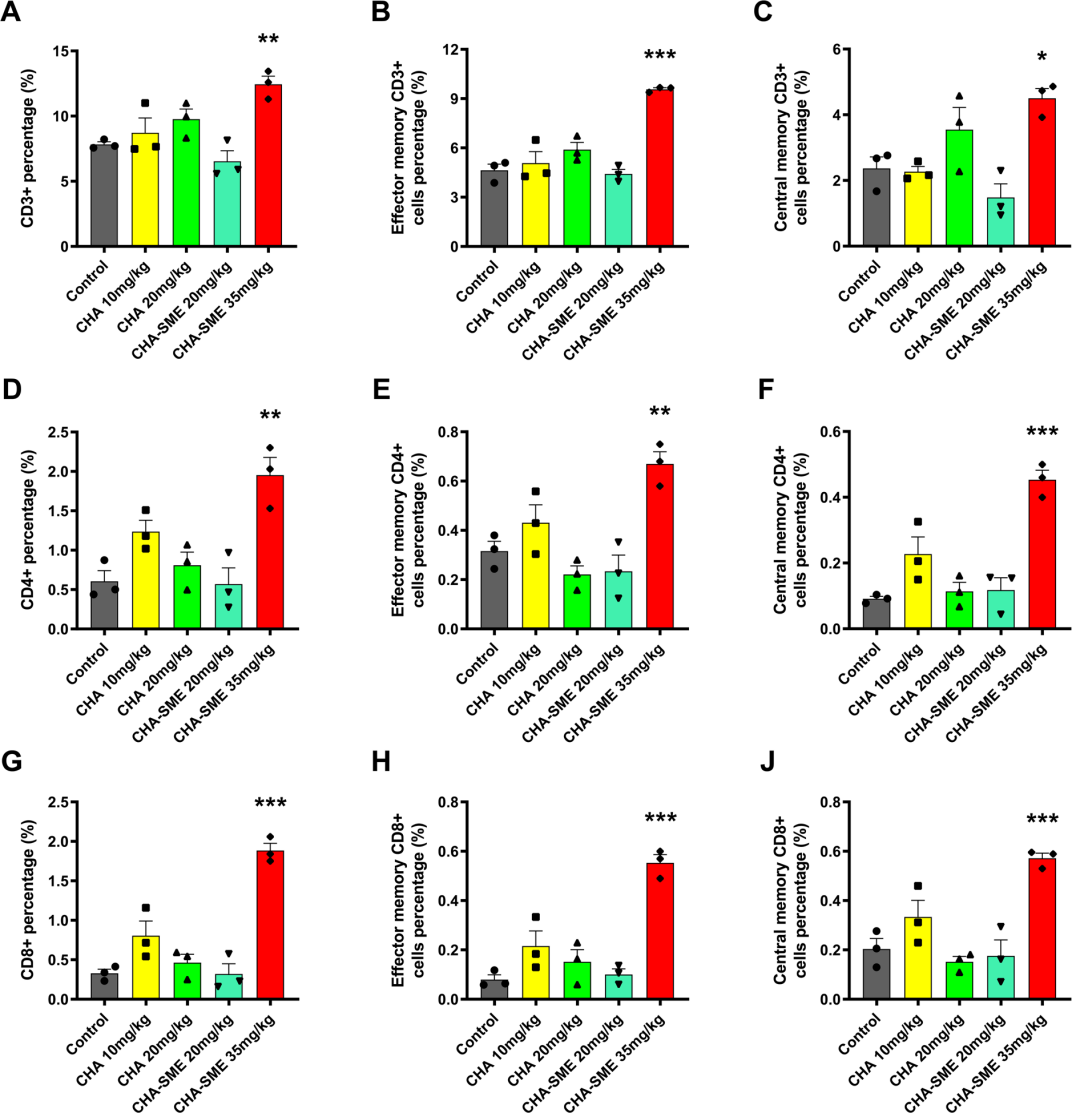
The effects of CHA-SME on the activation of T cells and the generation of memory T cells in tumor. (A–C) The percentage of CD3 +T cells in tumor and the percentage of T_EM_ and T_CM_ among CD3 +T cells. (E–G) The percentage of CD4+T cells in tumor and the percentage of TEM and TCM among CD4 +T cells. (H–J) The percentage of CD8 +T cells in tumor and the percentage of TEM and TCM among CD8 +T cells. Each value represents the mean±SEM (n=3). *P<0.05, **p<0.01, ***p<0.001 compared with the control group. CHA-SME, chlorogenic acid-encapsulated SMEDDS; T_CM_, central memory T cells; T_EM_, effector memory T cells.

The maturation of DCs is closely involved in their ability to prime naive T cells into effector T cells in the lymph nodes.^54^ The upregulation of classical phenotypic markers (MHC I, CD40, CD80, and CD86) on DCs suggested DCs maturation and the possibility of T-cell activation. Typically, MHC I-peptide complexes on the surface of DCs are presented to T cell receptor complexes on CD8 +T cells, which in turn promote T-cell activation, proliferation, and differentiation.^55^ Therefore, we quantified T cells in the MLNs, peripheral blood, spleen, and tumor by flow cytometry. Compared with the control group, although the proliferation of CD3+, CD4+, and CD8+T cells in the MLNs was nearly unchanged in the CHA-SME-exposed group, the proliferation of CD3+T cells in the peripheral blood and of CD4+ and CD8+T cells in the spleen was significantly increased after CHA-SME administration (online supplemental figures S7B and S8E, H). Activated T cells need to efficiently migrate from the MLNs and accumulate in the tumor microenvironment to induce antitumor immunity.^50 56^ Increased T-cell trafficking and infiltration in tumor sites leads to decreased tumor growth.^57^ Interestingly, CHA-SME (35 mg/kg) induced a significant increase in the proportion of inﬁltrating CD3+, CD4+, and CD8 +T cells into brain glioma tissues as compared with the control treatment, whereas no significant difference was detected in the CHA group (figure 8A, D G), corroborating the superiority of CHA-SME in T cell-mediated antitumor immunotherapy. The increase in brain glioma tissue-inﬁltrating T cells may be the result of activated T cells recruitment to tumors from the MLNs pool via the peripheral blood.^58 59^ As other important cellular immune-response markers, the levels of IFN-γ and TNF-α, which are associated with the activation of T cells, were measured to confirm the improvement of T-cell activity toward the tumor.^60^ As expected, CHA-SME promoted IFN-γ production in peripheral blood and spleens (online supplemental figure S9B, C). It was also noted that CHA-SME treatment led to substantially higher secreted levels of both IFN-γ and TNF-α in tumor tissues as compared with the control group (online supplemental figure S9D, H and K), confirming increased activation and infiltration of T cells into the tumor. In contrast to IFN-γ and TNF-α, the immunosuppressive cytokine IL-10 suppresses the activation of naive T cells and promotes the differentiation of immunosuppressive T-regulatory cells.^61 62^ As shown in online supplemental figure S9I, J, IL-10 level in the MLNs and peripheral blood was significantly reduced in the CHA-SME group compared with the control group. Our previous study found that CHA functions as an antitumor immunomodulator that promotes the polarization of tumor-associated macrophages from the M2 to the M1 phenotype via the promotion of STAT1 activation and the inhibition of STAT6 activation, thereby decreasing the secretion of IL-10 in both peripheral blood and tumor tissues.^20 23^ We speculate that CHA-SME treatment inhibits the expression of immunosuppressive cytokine IL-10 at least partially through the polarization of the M2 to the M1 phenotype macrophages. Our findings provided solid evidence that CHA-SME can promote the maturation of DCs and then prime the naive T cells into effector T cells, as well as inhibit the immunosuppressive component, thereby improving cancer immunotherapy outcomes. Additionally, CHA-SME may not be sufficient to induce the cytokine storm in vivo after consecutive administration for 14 days (online supplemental figure S10).

**Figure 9:**
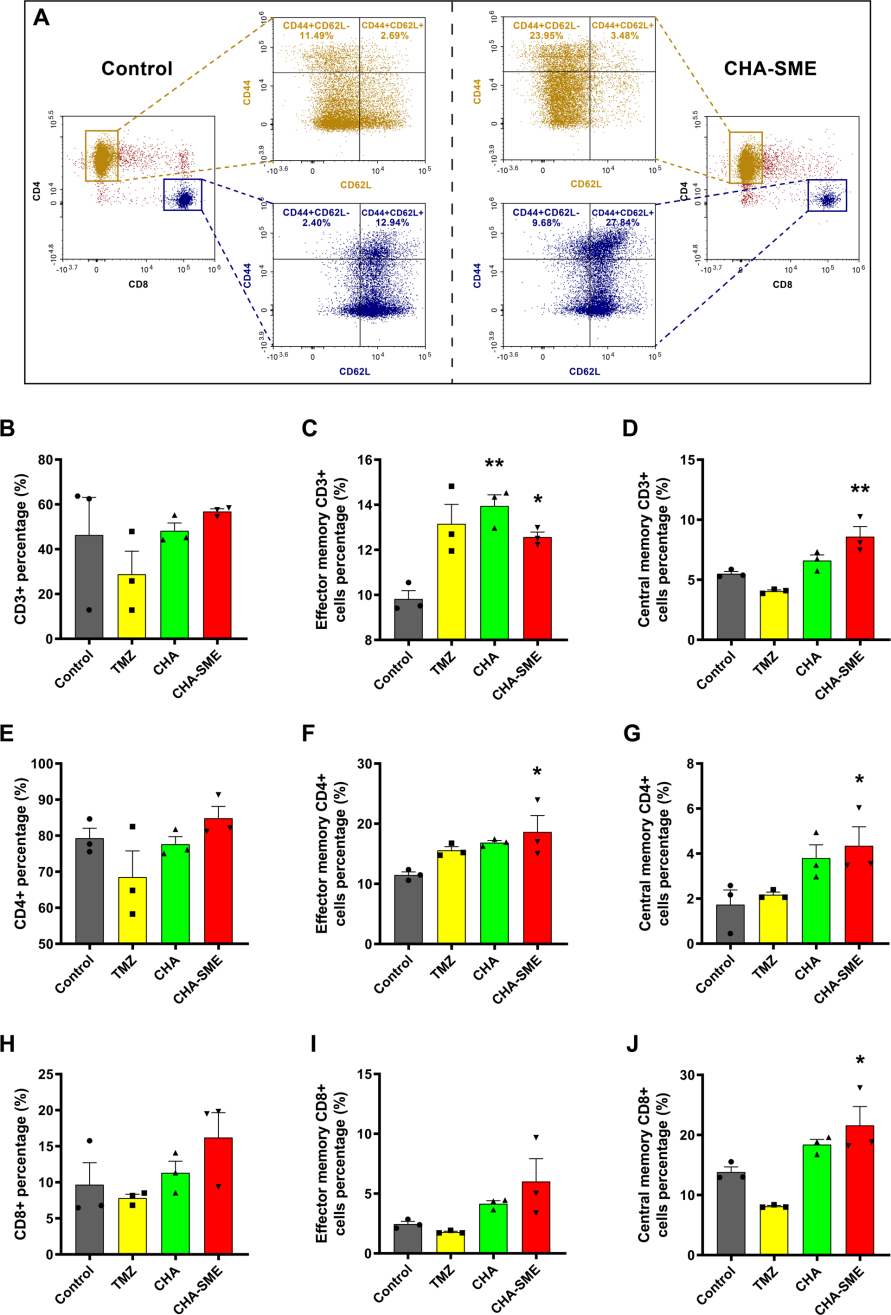
The effects of CHA-SME on the activation of T cells and the generation of memory T cells in MLNs. (A) Representative ﬂow cytometry analysis of CD4+, CD8+, and memory T cells in MLNs. (B–D) The percentage of CD3 +T cells in MLNs and the percentage of TEM and TCM among CD3 +T cells. (E–G) The percentage of CD4 +T cells in MLNs and the percentage of TEM and TCM among CD4 +T cells. (H–J) The percentage of CD8 +T cells in MLNs and the percentage of TEM and TCM among CD8 +T cells. Each value represents the mean±SEM (n=3). *P<0.05, **p<0.01 compared with the control group. MLNs, mesenteric lymph nodes; T_CM_, central memory T cells; T_EM_, effector memory T cells; TMZ, temozolomide.

In addition to the increased proportion of tumor-inﬁltrating T cells, another important characteristic of effective cancer immunotherapies is the memory feature, which confers a long-term antitumor response primarily based on the activity of memory T cells.^60^ The generation of memory T cells plays a critical role in the rapid clearance and neutralization of pathogens encountered previously by the immune system.^63^ Memory T cells can be typically classified into two subsets, T_CM_ and T_EM_, based on the phenotypic markers, functional attributes, and migratory properties. T_CM_ expressing CD62L preferentially migrate to the lymph nodes and possess strong recall responses, whereas T_EM_ lacking CD62L preferentially traffic to nonlymphoid tissues, exhibit cytotoxicity to target cells, and display a limited proliferative recall capacity.^64 65^ Thus, we quantified the proportions of T_CM_ and T_EM_ in vivo at the end of treatment. In the MLNs, the proportion of T_CM_ in CD3+ (CD45 +CD3+CD44+CD62L+), CD4+ (CD45 +CD3+CD4+CD44+CD62L+), and CD8+ (CD45 +CD3+CD8+CD44+CD62L+) T cells were significantly increased in mice treated with CHA-SME as compared with the control group (figure 9D, G, J). Additionally, CHA-SME showed significantly higher efficacy in promoting the expansion of T_EM_ in CD3+ (CD45 +CD3+CD44+CD62 L−), CD4+ (CD45 +CD3+CD4+CD44+CD62 L−), and CD8+ (CD45 +CD3+CD8+CD44+CD62 L−) T cells than the control group (figure 9C, F, I). Apart from the MLNs, the proportion of T_EM_ in CD3+, CD4+, and CD8 +T cells was also significantly elevated in the peripheral blood (online supplemental figure S7C, F, I) and spleen (online supplemental figure S8C, F, I) after CHA-SME administration. Interestingly, consistent with increased tumor-inﬁltrating T-cell fraction, CHA-SME significantly promoted the infiltration of both T_CM_ and T_EM_ into tumor sites as compared with the control treatment, whereas no significant differences were observed between the other groups and the control group (figure 8B–J). Taken together, these results indicate that CHA-SME can induce a long-term immune memory effect for immunotherapy.

### In vivo safety evaluation

**Figure 10:**
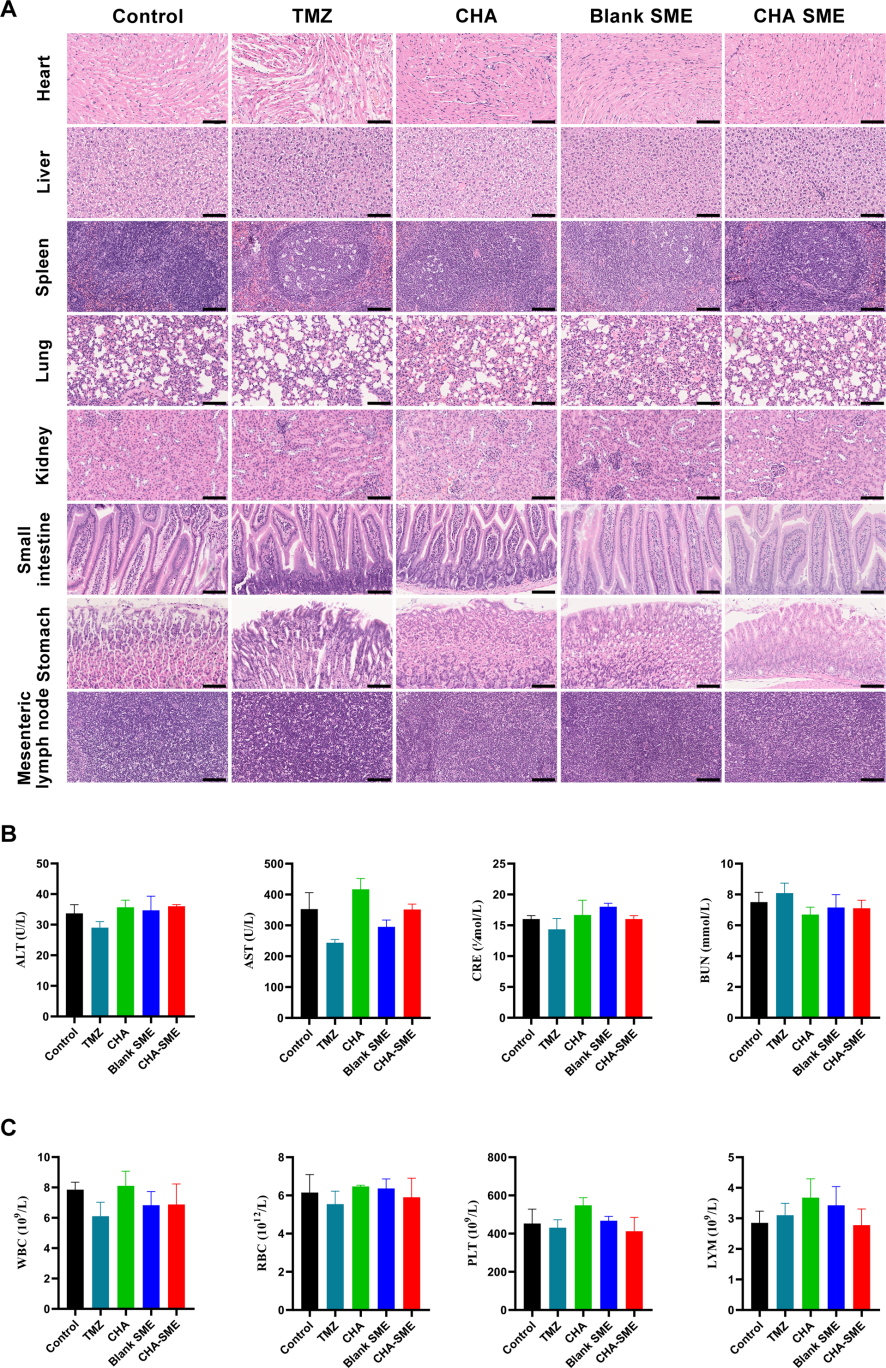
In vivo safety evaluation of CHA-SME. (A) Histopathologic analyses of H&E-stained tissue sections from heart, liver, spleen, lung, kidney, stomach, small intestine, and MLNs after the indicated treatment. Scale bar=100 µm. (B) The determination of blood biochemistry parameters including ALT, AST, CRE, and bun levels. Each value represents the mean±SEM (n=3). (C) The hematological examination, including the level of WBC, RBC, PLT, and LYM. Each value represents the mean±SEM (n=4). ALT, alkaline aminotransferase; AST, aspartate phosphatase; CHA-SME, chlorogenic acid-encapsulated SMEDDS; CRE, creatinine; LYM, lymphocyte; MLNs, mesenteric lymph nodes; PLT, platelet; RBC, red blood cells; TMZ, temozolomide; WBC, white blood cells.

The in vivo safety of CHA-SME was preliminarily evaluated by direct observations of animal behavior and body weight after administration. On chemotherapeutic agent TMZ administration, mice exhibited marked weight loss and sparse hair, which are common side effects of chemotherapy. However, all mice treated with free CHA and CHA-SME for 14 consecutive days tolerated the regimens well (figure 6D and online supplemental figure S6G). The safety of CHA-SME in the main organs was evaluated by histopathological and hematological examination. As shown in figure 10A, no obvious organ lesion (heart, liver, spleen, lung, and kidney) was observed in both the free CHA and CHA-SME groups as compared with the control group. However, mice treated with TMZ exhibited a certain degree of fragmentation and pathological changes in the heart and spleen. Similarly, there were no obvious organ lesions in the stomach, small intestine, and MLNs of CHA-SME-treated mice. Serum levels of ALT, AST, BUN, and CRE, which are biomarkers of liver and kidney toxicity, were not significantly different between the CHA-SME group and the control group, indicating no toxicity to the liver or kidney (figure 10B). No significant differences in hematological parameters, including the number of white blood cells, red blood cells, platelet, and LYM, were observed among all treated groups (figure 10C). Together, these results revealed that the immunotherapy of CHA-SME induces no systemic toxicity in vivo.

## Conclusion

In the study, we developed CHA-encapsulated SMEDDS to efficiently deliver CHA to the MLNs for glioblastoma immunotherapy. CHA-SME is highly capable of promoting drug accumulation within the MLNs via the lymphatic transport pathway and priming the naive T cells into effector T cells, thereby inhibiting glioma tumor growth. Therefore, oral CHA-SME presents a potential strategy for MLNs-targeted cancer immunotherapy of glioblastoma with circumventing poor penetration and drug resistance in immune checkpoint blockade therapy of glioblastoma. This study highlighted that promoting drug accumulation within the MLNs is a highly efﬁcient way to enhance LYM access and improved antitumor immune activity.

## Data Availability

All data relevant to the study are included in the article or uploaded as online supplemental information. The authors declare that all data supporting the results in this study are available within the paper and its supplementary information. Source data for the figures in this study are available from the corresponding author on reasonable request.
