# Supplementary material for: Oral SMEDDS promotes lymphatic transport and mesenteric lymph nodes target of chlorogenic acid for effective T-cell antitumor immunity
Source: J Immunother Cancer. 2021 Jul 16;9(7):e002753. doi: 10.1136/jitc-2021-002753 (PMC8287630; doi:10.1136/jitc-2021-002753)
Supplement: Supplementary data [file jitc-2021-002753supp001.pdf]

Original Article

Oral SMEDDS Promotes Lymphatic Transport and Mesenteric Lymph Nodes Target of Chlorogenic Acid for Effective T-cell Antitumor Immunity

Jun Ye[a,b], Yue Gao[a,b], Ming Ji[a], Yanfang Yang[a,b], Zhaohui Wang [a,b], Baolian Wang[a], Jing Jin[a], Ling Li[a], Hongliang Wang[a,b], Xiaoyan Xu[a,b], Hengfeng Liao[a,b], Chunfang Lian[a,b], Yaqi Xu[a,b], Renjie Li[a,b], Tong Sun[a,b], Yan Li[a], LiliGao [a,b], Xiaoguang Chen[a*], and Yuling Liu[a,b*]

[a]State Key Laboratory of Bioactive Substance and Function of Natural Medicines, Institute of Materia Medica, Chinese Academy of Medical Sciences & Peking Union Medical College, Beijing 100050, P.R. China

[b]Beijing Key Laboratory of Drug Delivery Technology and Novel Formulation, Institute of Materia Medica, Chinese Academy of Medical Sciences & Peking Union Medical College, Beijing 100050, P.R. China

Correspondence: Professor Xiaoguang Chen and Professor Yuling Liu, State Key Laboratory of Bioactive Substance and Function of Natural Medicines, Institute of Materia Medica, Chinese Academy of Medical Sciences & Peking Union Medical College, 1 Xiannongtan Street, Beijing 100050, P.R. China, Tel (86)10-89285188, Fax (86)10-89285190, Email chxg@imm.ac.cn (Xiaoguang Chen), ylliu@imm.ac.cn (Yuling Liu).

Running title: SMEDDS Promotes Lymphatic Transport for Effective T-cell Immunity

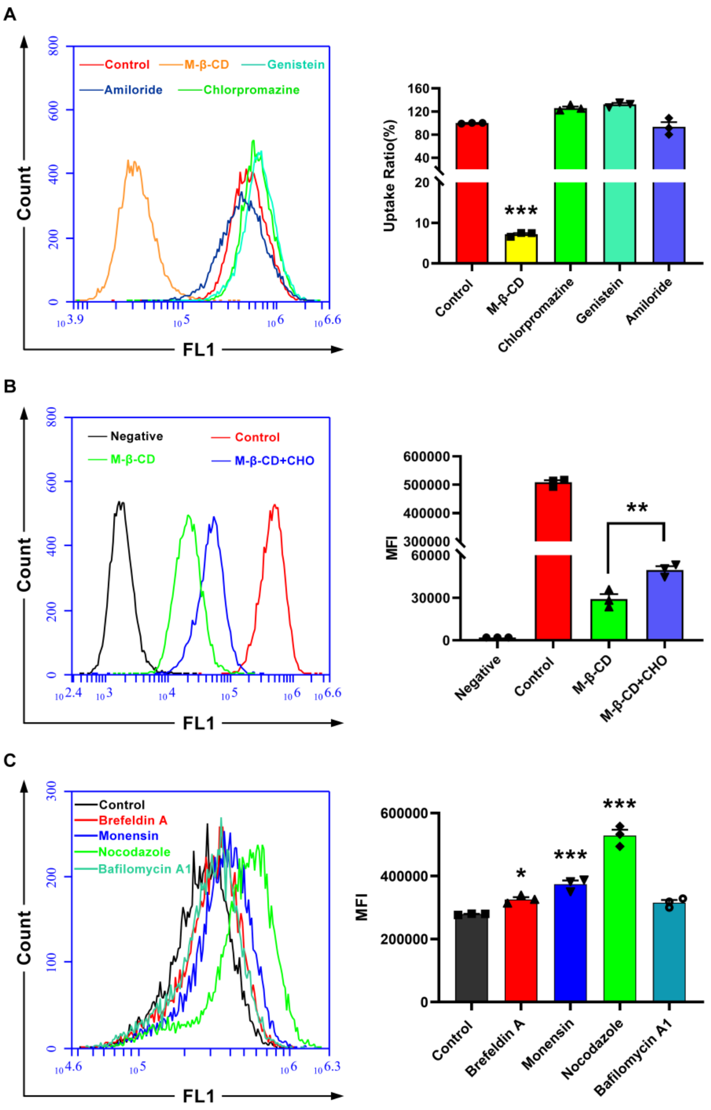

Fig. S1 Endocytosis and exocytosis pathways of CHA-SME in Caco-2 cells. (A) The influence of different endocytosis inhibitors on the endocytosis of CHA-SME monitored by flow cytometry. Each value represents the mean ± SEM (n = 3). ***p < 0.001 compared with the control group. (B) The effect of cholesterol on the endocytosis of CHA-SME monitored by flow cytometry. Each value represents the mean ± SEM (n = 3). **p < 0.01. (C) The influence of various endocellular transport inhibitors on the exocytosis of CHA-SME monitored by flow cytometry. Each value represents the mean ± SEM (n = 3). *p < 0.05 and ***p < 0.001 compared with the control group.

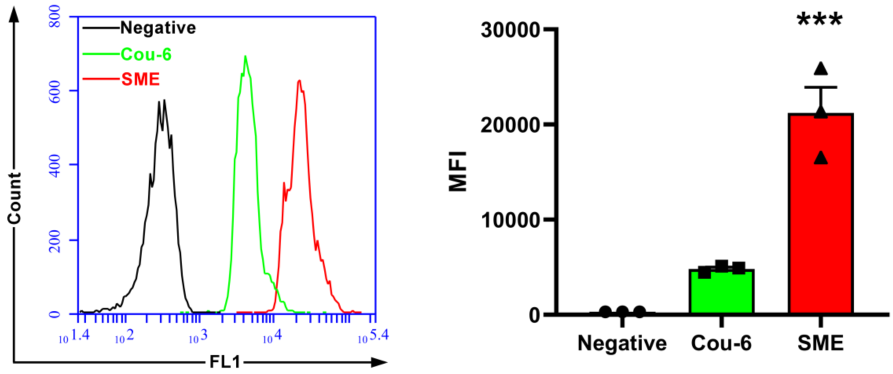

Fig. S2 The in vitro cellular uptake profile of Cou-6-labeled CHA-SME in lymphocytes derived from MLNs determined by flow cytometry. Each value represents the mean ± SEM (n = 3). ***p < 0.001 compared with the Cou-6.

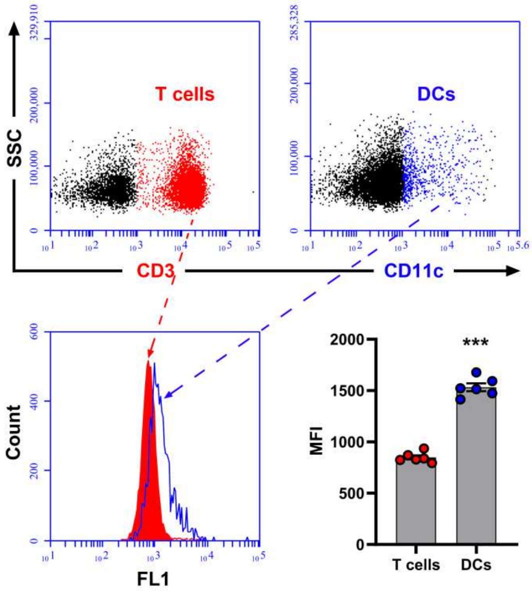

Fig. S3 The in vitro cellular uptake profile of Cou-6-labeled CHA-SME in lymphocytes derived from MLNs determined by flow cytometry. The lymphocytes derived from MLNs were stained with CD3 and CD11c, which represented as T cells and DCs, respectively. Each value represents the mean ± SEM (n = 6). ***p < 0.001.

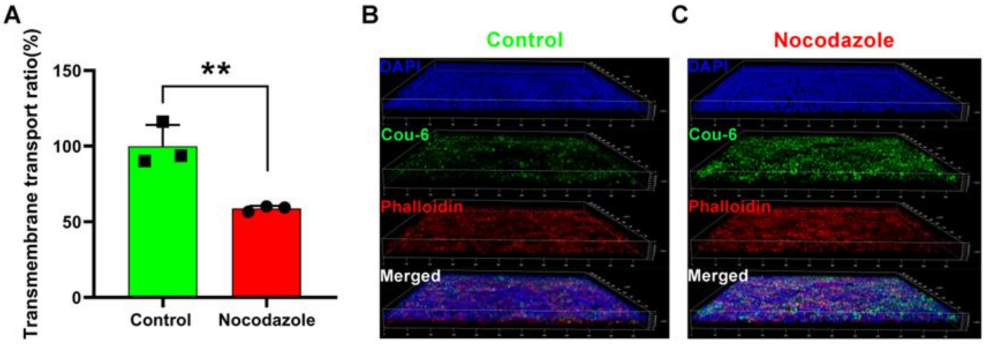

Fig. S4 Transport mechanism of CHA-SME across Caco-2 cell monolayers. (A) The impact of the microtubule inhibitor nocodazole on the CHA-SME transportation across Caco-2 cell monolayers. Each value represents the mean ± SEM (n = 3). **p < 0.01. (B and C) CLSM images of Caco-2 cell monolayer. The Caco-2 cell monolayer was treated with Cou-6-labeled CHA-SME, washed with cold HBSS, and then incubated with the microtubule inhibitor nocodazole. The cell nuclei and cytoskeleton were stained with DAPI (blue) and phalloidin (red), respectively.

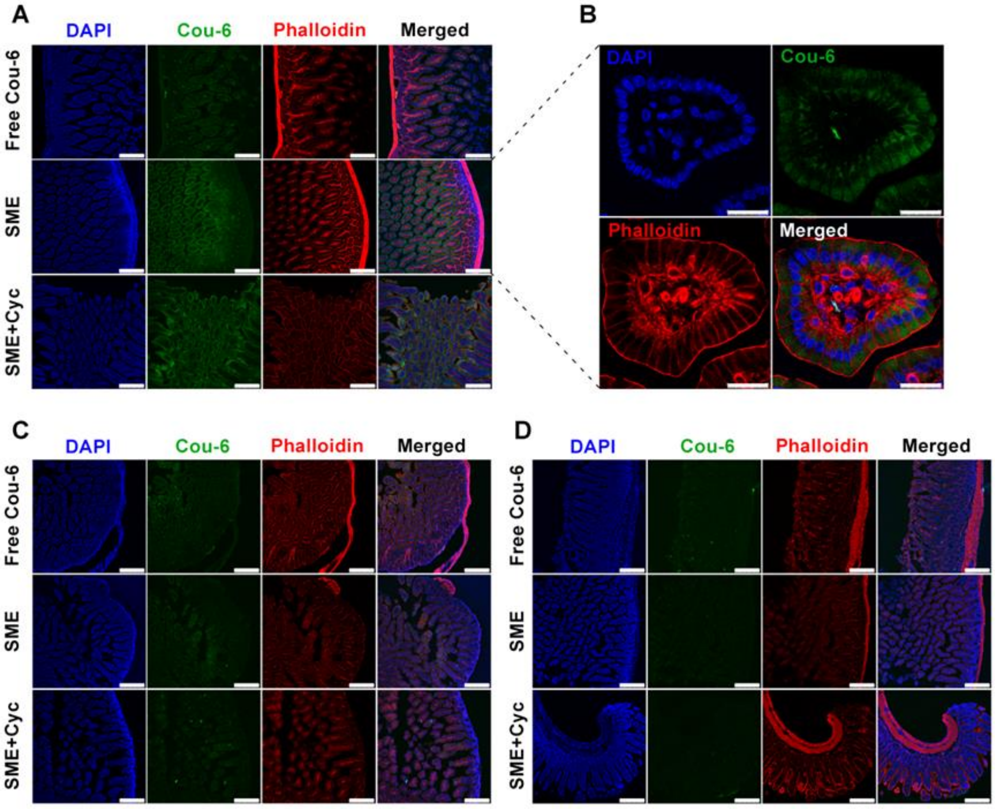

Fig. S5 The absorption of Cou-6-labeled CHA-SME in the intestinal tract after oral administration. (A) CLSM images of duodenum frozen tissue sections. Scale bar = 250 μm. (B) Representative magnification view of duodenum frozen tissue sections of Cou-6-labeled CHA-SME-treated mice. Scale bar = 25 μm (C) CLSM images of jejunum frozen tissue sections. Scale bar = 250 μm. (D) CLSM images of ileum frozen tissue sections. Scale bar = 250 μm. The cell nuclei and cytoskeleton were stained with DAPI (blue) and phalloidin (red), respectively. “SME” represents the mice that are orally administered Cou-6-labeled CHA-SME. “SME+Cyc” represents the mice that are pretreated with cycloheximide and orally administered Cou-6-labeled CHA-SME.

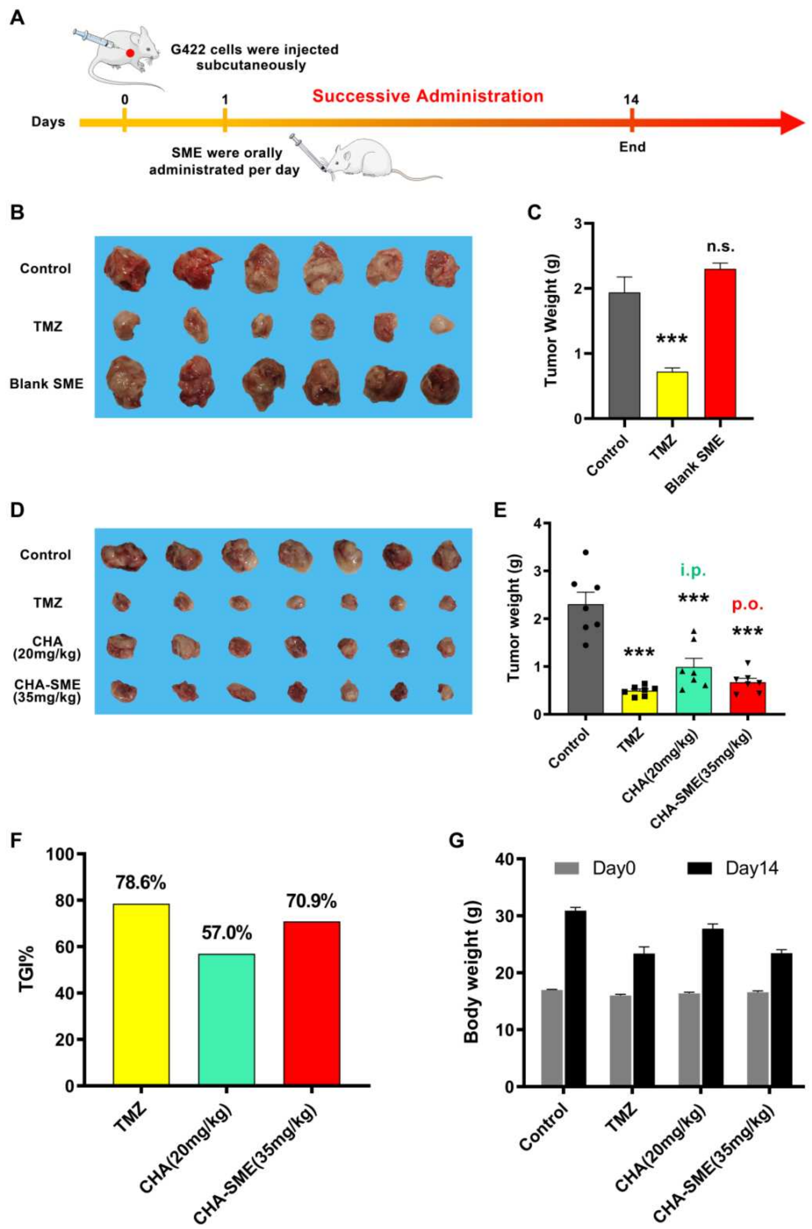

Fig. S6 In vivo antitumor efficacy of CHA-SME in a subcutaneous G422 glioma murine model. (A) The schematic of treatment schedule. The images of excised tumors (B) and tumor weight (C) of subcutaneous murine G422 glioma tumor-bearing mice after treatment of blank CHA-SME. Each value represents the mean ± SEM (n = 6).The images of excised tumors (D), tumor weight (E), tumor growth inhibition ratio (TGI%) (F), and body weight changes (G) of subcutaneous murine G422 glioma tumor-bearing mice after treatment of CHA-SME. Each value represents the mean ± SEM (n = 7). n.s., not significant. ***p < 0.001 compared with the control group. i.p., intraperitoneally; p.o., orally.

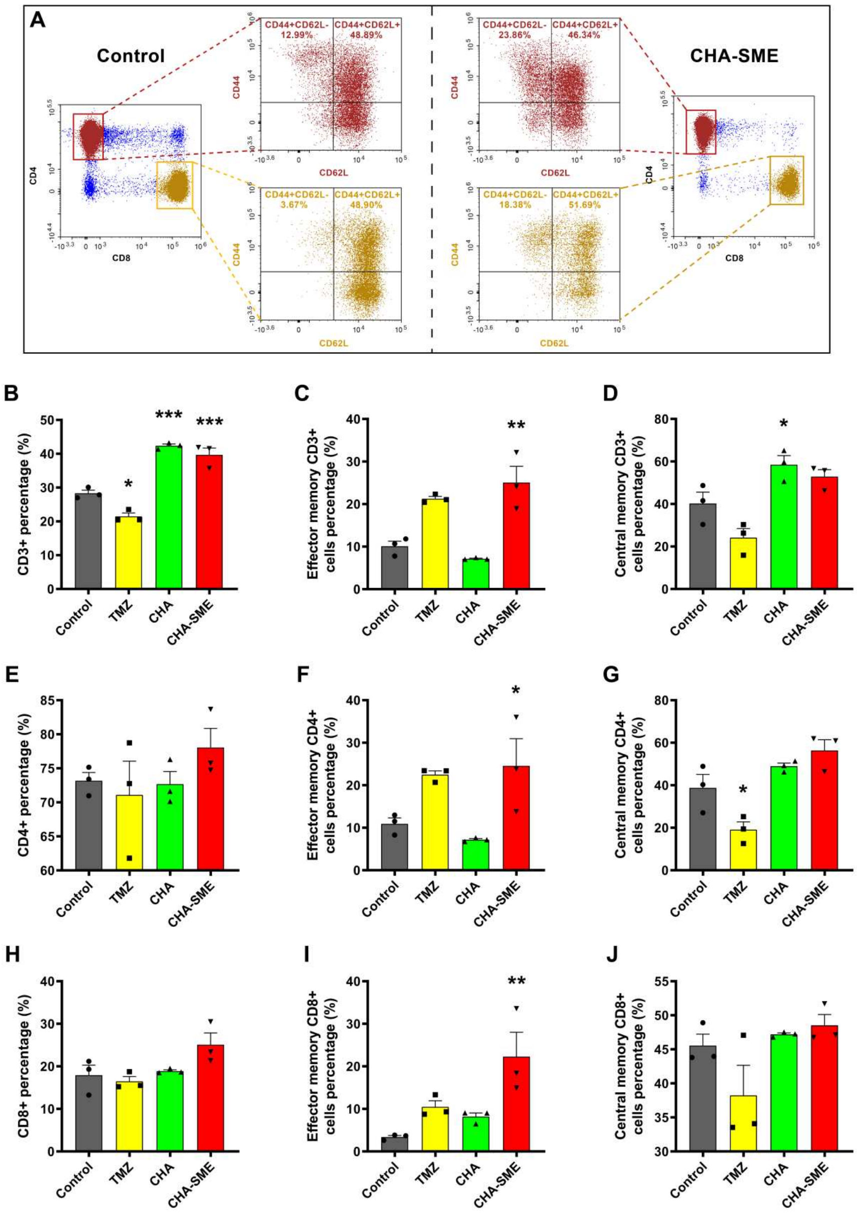

Fig. S7 The effects of CHA-SME on the activation of T cells and the generation of memory T cells in peripheral blood. (A) Representative flow cytometry analysis of CD4+, CD8+, and memory T cells in peripheral blood. (B–D) The percentage of CD3+ T cells in peripheral blood and the percentage of T_{EM} and T_{CM} among CD3+ T cells. (E–G) The percentage of CD4+ T cells in peripheral blood and the percentage of T_{EM} and T_{CM} among CD4+ T cells. (H–J) The percentage of CD8+ T cells in peripheral blood and the percentage of T_{EM} and T_{CM} among CD8+ T cells. Each value represents the mean ± SEM (n = 3). *p < 0.05, **p < 0.01, and ***p < 0.001 compared with the control group.

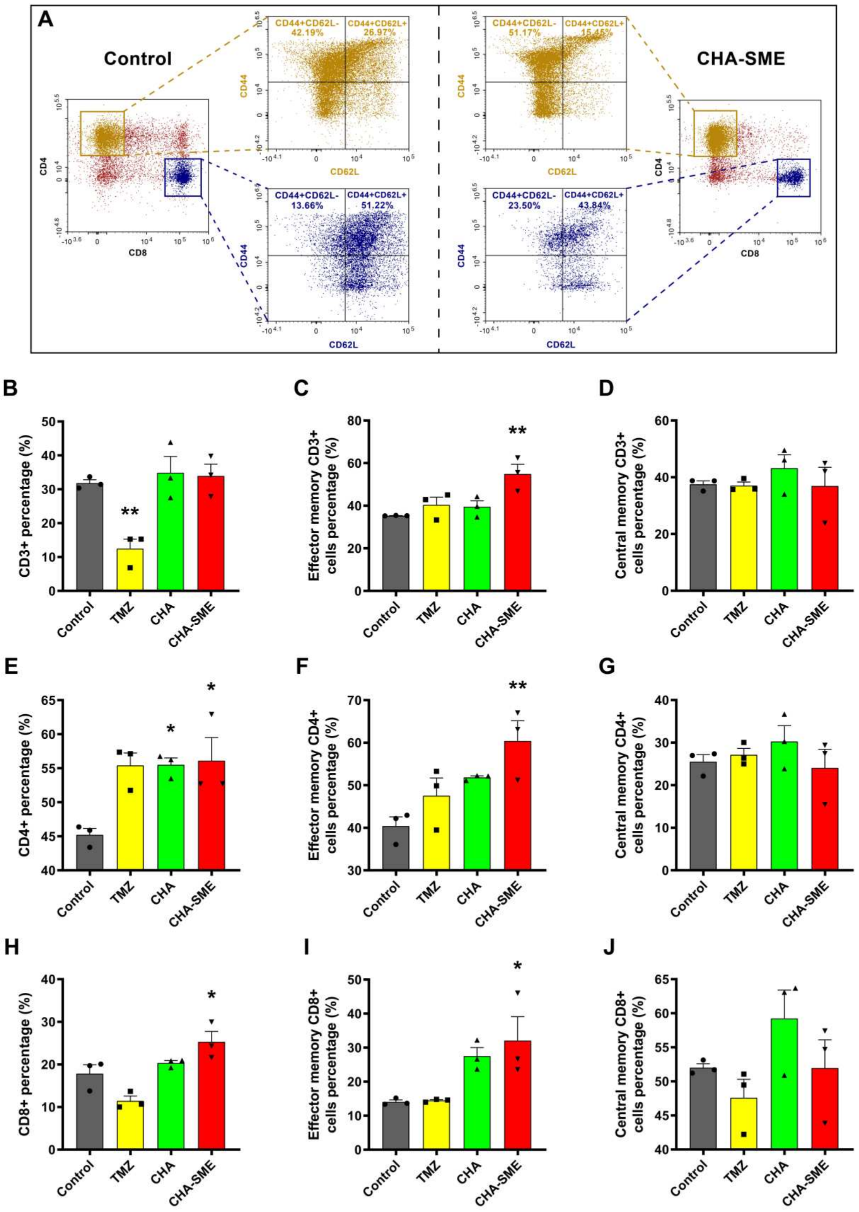

Fig. S8 The effects of CHA-SME on the activation of T cells and the generation of memory T cells in spleen. (A) Representative flow cytometry analysis of CD4+, CD8+, and memory T cells in spleen. (B–D) The percentage of CD3+ T cells in spleen and the percentage of T_{EM} and T_{CM} among CD3+ T cells. (E–G) The percentage of CD4+ T cells in spleen and the percentage of T_{EM} and T_{CM} among CD4+ T cells. (H–J) The percentage of CD8+ T cells in spleen and the percentage of T_{EM} and T_{CM} among CD8+ T cells. Each value represents the mean ± SEM (n = 3). *p < 0.05 and **p < 0.01 compared with the control group.

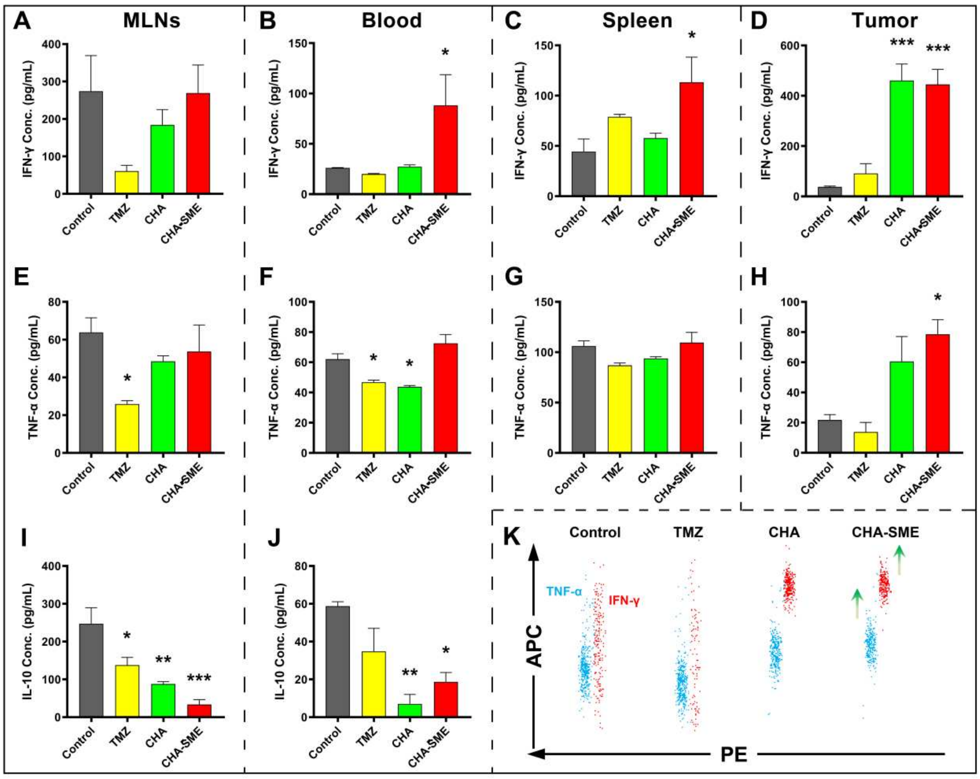

Fig. S9 The cytokine production. Cytokine secretion of IFN-γ in MLNs (A), blood (B), spleen (C), and tumor (D). Cytokine secretion of TNF-α in MLNs (E), blood (F), spleen (G), and tumor (H). Cytokine secretion of IL-10 in MLNs (I) and blood (J). (K) Flow cytometric dot plots of cytokine production of IFN-γ and TNF-α induced by CHA-SME in tumor. Each value represents the mean ± SEM (n = 3). *p < 0.05, **p < 0.01, and ***p < 0.001 compared with the control group.

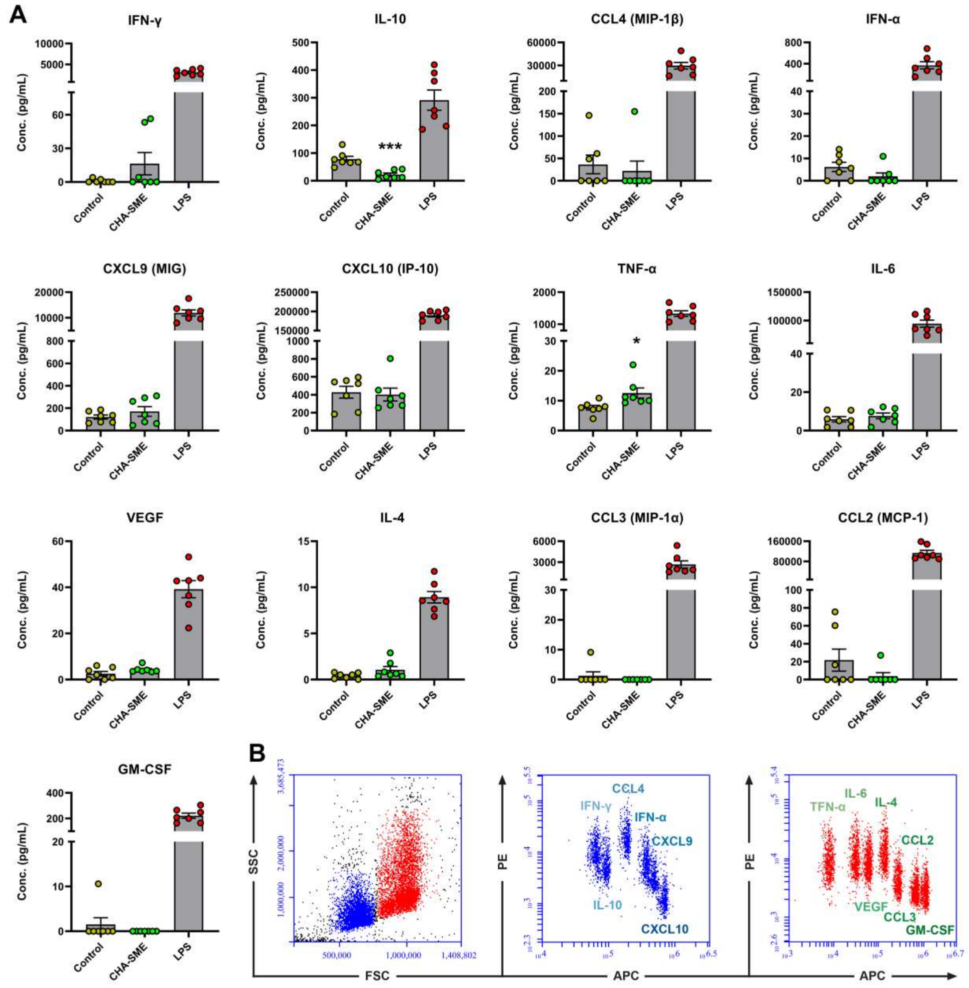

Fig. S10 (A) 13 key biomarkers involved in cytokine release syndrome such as IFN-γ, IL-10, CCL4 (MIP-1β), IFN-α, CXCL9 (MIG), CXCL10 (IP-10), TNF-α, IL-6, VEGF, IL-4, CCL3 (MIP-1α), CCL2 (MCP-1), and GM-CSF in peripheral blood were quantitatively determined using bead-based LEGENDplex™ Mouse Cytokine Release Syndrome Panel. (B) Representative flow cytometric dot plots of cytokine determination. Each value represents the mean ± SEM (n = 7). *p < 0.05 and ***p < 0.001 compared with the control group.
